# Association Between Sex and Mortality After Traumatic Brain Injury: A Systematic Review and Meta-Analysis

**DOI:** 10.1007/s12028-026-02521-3

**Published:** 2026-04-24

**Authors:** Elise Beijer, Floor J. Mansvelder, Romein W. G. Dujardin, Nicole P. Juffermans, Linda J. Schoonmade, Leo M. G. Geeraedts, Frank W. Bloemers, Charissa E. van den Brom, Patrick Schober

**Affiliations:** 1 Department of Surgery, Section Trauma Surgery, Amsterdam University Medical Centers, VU University Amsterdam, de Boelelaan 1117, Amsterdam, The Netherlands; 2 Department of Anesthesiology, Amsterdam University Medical Centers, VU University Amsterdam, de Boelelaan 1117, 1105 AZ Amsterdam, The Netherlands; 3 Laboratory of Experimental Intensive Care and Anesthesiology (LEICA), Amsterdam University Medical Centers, University of Amsterdam, Amsterdam, The Netherlands; 4 Department of Intensive Care Medicine, Erasmus MC, University Medical Center Rotterdam, Dr. Molewaterplein 40, Rotterdam, The Netherlands; 5 Medical Library, VU University Amsterdam, Amsterdam, The Netherlands; 6 Department of Intensive Care Medicine, Amsterdam University Medical Centers, University of Amsterdam, Amsterdam, The Netherlands; 7 Helicopter Emergency Medical Service Lifeliner 1, Westelijk Havengebied, Amsterdam, The Netherlands

**Keywords:** Brain injuries, Trauma, Nervous system, Wounds and injuries, Sex differences, Mortality

## Abstract

Traumatic brain injury (TBI) is a major cause of trauma-related mortality. While male individuals are disproportionately affected, the influence of sex on mortality remains controversial, with conflicting evidence suggesting female advantage, disadvantage, or no difference. Clarifying this relationship is crucial for prognosis and sex-specific treatment. A systematic search was conducted on 4 June 2025 in PubMed, Embase, Web of Science, and the Cochrane Library without date restrictions. Observational studies reporting sex-stratified TBI patient mortality with sufficient data to derive effect sizes were included. Screening, full-text assessment, and data extraction were performed independently by multiple reviewers. Random-effects meta-analysis was conducted to calculate odds ratios (OR) for mortality in female versus male individuals, with subgroup and leave-one-out sensitivity analyses as well as meta-regression. Heterogeneity was quantified using *I*^2^ and prediction intervals. Quality was assessed using an adapted Newcastle–Ottawa Scale. The review followed Preferred Reporting Items for Systematic Reviews and Meta-Analyses (PRISMA) and Meta-Analysis of Observational Studies in Epidemiology (MOOSE) guidelines. A total of 40 studies, encompassing over 1 million patients (383,914 female individuals; 662,748 male individuals), were included. Overall, sex was not significantly associated with TBI mortality [OR 0.99; 95% confidence interval (CI) 0.90–1.09], though substantial between-study heterogeneity and prediction intervals indicated that female mortality could be higher in some contexts and lower in others. Subgroup analyses showed no significant differences by TBI type, age, or severity. Although initial analyses suggested lower female mortality in good-quality studies and higher female mortality in patients with severe TBI and in studies conducted before the 2000s, the findings were not robust in leave-one-out analyses. Meta-regression indicated that mean age differences did not explain heterogeneity between sex and mortality estimates. Concluding, there is no universal biological association between sex and TBI mortality. Instead, the relationship appears context-dependent, varying by study characteristics and patient populations. Future research should move beyond assessing sex as a simplistic risk factor and focus on identifying specific biological and clinical contexts where sex influences outcomes to inform personalized care.

**Supplementary Information:**

The online version contains supplementary material available at 10.1007/s12028-026-02521-3.

## Introduction

Traumatic injuries cause roughly 4.4 million deaths each year [1, 2], with traumatic brain injury (TBI) accounting for nearly 2 million [3, 4], representing a major source of trauma-related mortality and neurological morbidity [5].

Trauma-related research is challenging owing to heterogeneity in the study population. Numerous factors may influence patient outcomes, including age, injury severity and mechanism, and potentially sex. Epidemiological data show distinct sex differences in the incidence of trauma, with male individuals accounting for approximately 70% of all TBI cases [6, 7]. The role of sex in outcomes following TBI has been examined in various clinical and preclinical studies, but findings are conflicting [8–12]. Clinical studies are heterogenous. Sex-specific differences in immune and organ system responses are suggested; yet mortality data are inconsistent, with reports of both worse and better outcomes for female individuals [12–15]. Preclinical evidence often favors female individuals, with administration of exogenous estrogen demonstrating lower mortality and less complications in rodents models [16–18].

Given the conflicting and inconclusive literature about the nature and strength of the relationship between sex and mortality in TBI populations, we performed a systematic review and meta-analysis to clarify this association.

## Methods

### Protocol and Registration

This systematic review and meta-analysis adheres to the Preferred Reporting Items for Systematic Reviews and Meta-Analyses (PRISMA) [19, 20] and Meta-Analysis of Observational Studies in Epidemiology (MOOSE) guidelines [21]. Methods were specified a priori in a study protocol [22]. The study was registered at the International Prospective Register of Systematic Reviews (PROSPERO) with number CRD42021234582.

### Eligibility Criteria

Studies were included if they: (1) reported sex-related differences, (2) included human patients with physical traumatic injury, and (3) reported mortality outcomes with sufficient data to calculate an effect size. We excluded studies if: (1) no full text was available, (2) they were not written in English, (3) they included only burns, snake bites, substance ingestion, and/or poisoning, (4) they were a meta-analysis, review, editorial, experimental study, discussion, letter, case report, or conference abstract, or (5) they used a specific intervention or protocol that could influence mortality rates.

### Information Sources and Search Strategy

Four bibliographic databases (PubMed, Embase, Clarivate Analytics/Web of Science Core Collection, and the Wiley/Cochrane Library) were systematically searched in collaboration with a medical information specialist (L.S.) from inception to 4 June 2025. No methodological filters or date restrictions were applied. Search terms included index terms and free-text words, covering synonyms and related concepts for traumatic injury and sex-related mortality differences. The full search strategy is detailed in Online Appendix A. Duplicate articles were excluded using Endnote X21.5, AED method [23] and Bramer method [24]. Google Scholar and reference lists were searched for additional relevant literature.

The initial search targeted the overall trauma population; however, prior to any formal analyses, we narrowed the focus to TBI because of the large number of studies, and the broad scope and heterogeneity of trauma. Because TBI represents a distinct clinical entity, we determined that pooling TBI data with other traumatic injuries would limit the clinical interpretability of the findings. Studies meeting the inclusion criteria but unrelated to TBI will be addressed separately.

Three reviewers (E.B., R.D., and F.M.) independently screened titles and abstracts for eligibility using Rayyan [25]. Full texts of potentially relevant records were retrieved and independently screened by two reviewers (E.B. and F.M.). Disagreements were resolved through consensus, or, if needed, a third reviewer (P.S.).

### Data Extraction

Data were extracted by one reviewer (E.B.) using a standardized pre-piloted data collection form and checked by a second reviewer (F.M.). Data extraction included: (1) study characteristics (author, year, country, design, period, inclusion/exclusion criteria, population, and sex-specific sample sizes; (2) patient and injury characteristics (age, severity, and mechanism); and (3) mortality outcomes (crude and when available adjusted effect sizes with corresponding confidence intervals and *p*-values). Discrepancies were resolved through discussion, or by consulting a third reviewer (P.S.).

### Assessment of Study Quality and Risk of Bias

Two authors (E.B. and F.M.) independently assessed quality, with a third author (P.S.) resolving disagreements. We used the Newcastle–Ottawa scale (NOS) to assess methodological quality [26]. Based on Lui et al. [27], we adapted the NOS by adding two domains (population size and study design). Furthermore, we adapted the comparability domain to align with our objective to assess the total association between sex and mortality after TBI. Because factors such as injury severity and injury mechanism may be downstream variables that mediate this relationship, statistical adjustment for potential intermediates could attenuate estimates of the total sex–mortality association [28]. In addition, adjusted effect estimates were inconsistently reported across studies and based on varying covariate sets that target different estimands. Therefore, in the comparability domain, we did not score studies on the basis of statistical adjustment but instead prioritized transparent reporting of sex-stratified baseline differences. The adapted NOS criteria are provided in Online Appendix B.

### Data Synthesis and Statistical Analysis

The primary measure was the odds ratio (OR) for mortality in female versus male patients with TBI. A random-effects meta-analysis was performed with STATA 19.0 (StataCorp, College Station, TX, USA) to allow for between-study heterogeneity [29]. Pooled effect sizes and 95% confidence intervals (CI) were derived from crude mortality data. To avoid duplicate patient inclusion, the analysis providing the most precise estimate (smallest standard error) was selected in case of overlapping datasets.

All types of TBI and mortality measures were initially pooled to provide an overall estimate. In sensitivity analyses, we explored the influence of methodological quality and timing of mortality assessment. We additionally performed leave-one-out sensitivity analyses to assess the robustness of the pooled estimates and identify whether any single study disproportionately influenced the results. We also stratified by isolated/mixed TBI, TBI severity, age groups, and study period (defined by the calendar era encompassing the majority of data collection). Differences between subgroups were assessed using Cochran’s *Q* test, with a *p*-value < 0.05 considered statistically significant. Relative heterogeneity was quantified using the *I*^2^ statistic [30, 31], whereas 95% prediction intervals (PI) were calculated as an absolute measure of heterogeneity. [32]

To explore whether sex-specific age imbalance across studies influenced the observed association between sex and mortality, we performed a random-effects meta-regression using the difference in mean age between female and male patients as a study-level moderator. For studies reporting age as median with interquartile range, means were estimated using the method described by Wan et al. [33]

To assess small-study bias, we plotted the natural logarithm of the odds ratio against its standard error and evaluated funnel-plot asymmetry using visual inspection and Egger’s regression test [34].

## Results

### Study Selection

**Fig. 1:**
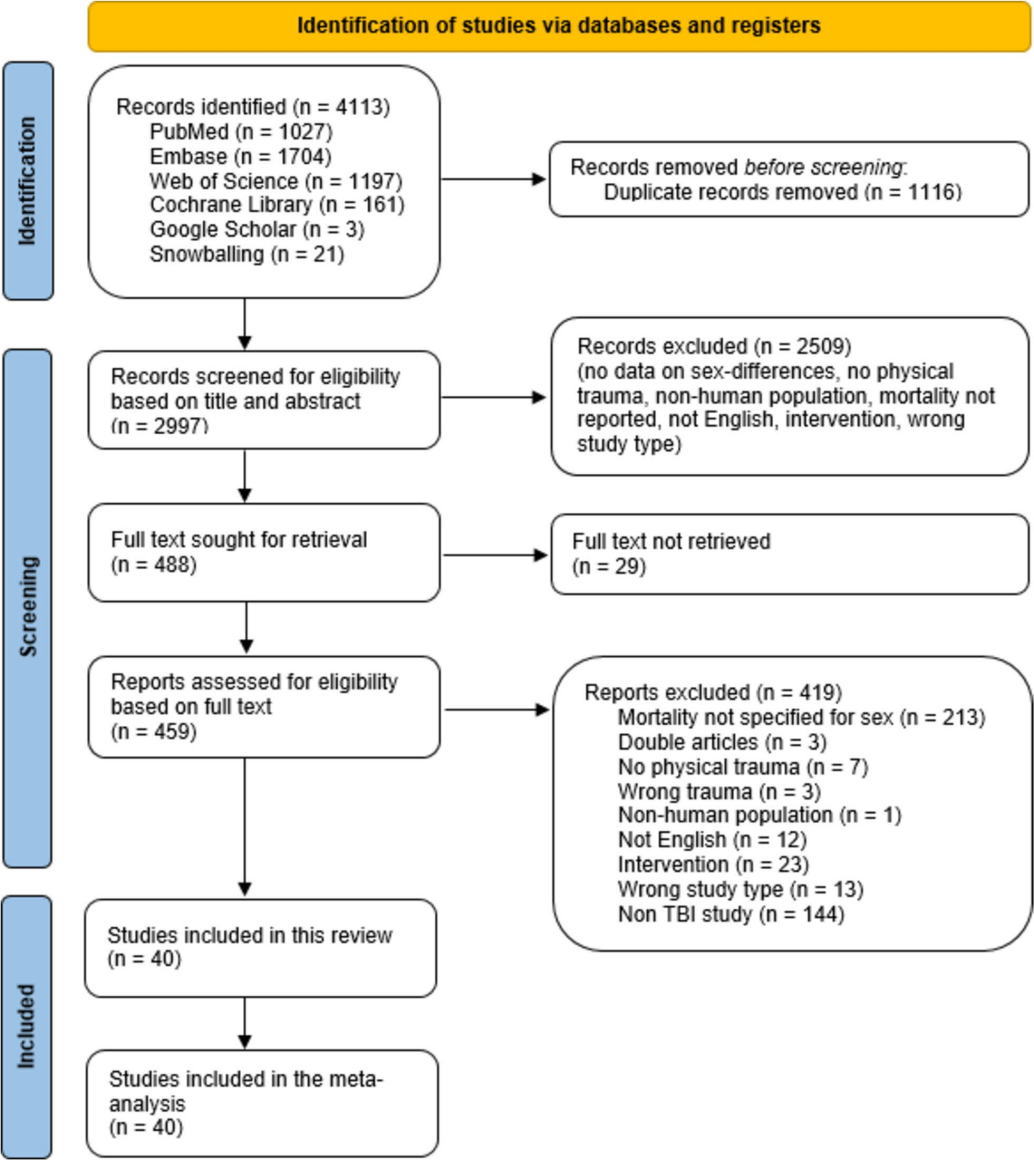
PRISMA flow diagram. prisma flow diagram summarizing identification, screening, eligibility, and inclusion of studies

The literature search identified 4113 records. After deduplication and screening, 459 full-text articles were assessed for eligibility. Of these, 419 did not meet the inclusion criteria, resulting in the inclusion of 40 studies [35–74]. The flow chart of the search and selection process is shown in Fig. 1 [75].

### Study Characteristics

The 40 included studies were published from 1981 to 2024 and comprised data from over 1 million patients (383,914 female and 662,748 male individuals). Most studies were conducted in North America (*n* = 18), Europe (*n* = 9), and Asia (*n* = 9), with the remainder from Oceania, the Middle East, and South America. A total of 16 studies were retrospective cohort studies, 14 were registry-based retrospective analyses, and 10 were prospective cohort studies (Supplementary Table 1). The NOS rating ranged between 6 and 11 stars, with a median of 7 (Table 1).

**Table 1:** Quality assessment

Author(s)	Year	Se	C	O	Si	St	☆	Quality
Ahmed	2020	4	0	3	1	1	9	Poor
Albrecht	2016	4	2	2	0	0	8	Poor
Beijer	2023	4	2	3	0	0	9	Poor
Berry	2009	4	2	3	1	0	10	Good
Chinese Head Trauma Study Collaborators	2021	4	0	2	1	0	7	Poor
Coimbra	2003	4	2	2	0	0	8	Poor
Colantonio	2008	4	0	2	0	0	6	Poor
Davis	2006	4	0	2	1	0	7	Poor
de Guise	2014	4	1	3	1	0	9	Good
Elkbuli	2020	4	2	2	0	0	8	Poor
El-Menyar	2022	4	2	3	1	0	10	Good
Falk	2015	4	0	2	0	0	6	Poor
Gan	2004	4	0	3	0	0	7	Poor
Gao	2017	4	0	2	0	0	6	Poor
Gujral	2006	4	0	2	1	0	7	Poor
Herrera-Melero	2015	4	1	2	0	0	7	Poor
Hong	2020	4	1	2	1	0	8	Good
Hosomi	2021	4	2	3	1	1	11	Good
Jung	2024	4	1	2	0	1	8	Fair
Kadar	2019	4	0	2	0	0	6	Poor
Khan	2019	4	0	2	0	1	7	Poor
Kirkness	2004	4	1	3	0	1	9	Fair
Klauber	1981	4	0	2	1	1	8	Poor
Kokkinou	2020	4	0	2	0	0	6	Poor
Kraus	2000	4	0	2	0	1	7	Poor
Leitgeb	2011	4	2	3	0	1	10	Fair
Ley	2013	4	1	2	1	0	8	Good
Mair	2022	4	2	3	1	1	11	Good
Martins	2009	4	0	2	0	1	7	Poor
Mikolić	2021	4	1	3	1	1	10	Good
Morrison	2004	4	0	2	1	0	7	Poor
Munivenkatappa	2016	4	0	3	0	0	7	Poor
Ng	2006	4	1	2	0	1	8	Fair
O’Reilly	2023	4	0	2	1	0	7	Poor
Ottochian	2009	4	1	2	0	0	7	Poor
Rappold	2002	4	1	2	1	0	8	Good
Saatian	2018	4	0	2	1	0	7	Poor
Shibahashi	2017	4	0	2	1	0	7	Poor
Svedung Wettervik	2022	4	0	3	0	0	7	Poor
Yeung (AUS)	2011	4	1	2	0	0	7	Poor
Yeung (CHINA)	2011	4	1	2	0	0	7	Poor

Because study quality was assessed using categorical ratings, studies with the same number of allocated stars could still differ in overall quality classification (poor, fair, or good)

Se, selection (max. 4☆); C, comparability (max. 2☆); O, outcome (max. 3☆); Si, size (max. 1☆); St, study design (max. 1☆)

### Patient and Injury Characteristics: Age and Type, Severity, and Mechanism of Injury

Participants were predominantly male (Supplementary Table 1). Age distributions varied across studies; 18 focused on adults, 3 on older patients (≥ 65 years), and 2 on pediatric populations only, while others applied no age restrictions. Generally, female patients tended to be older than male patients. Regarding TBI type, 11 studies specifically researched isolated TBI, while others included mixed TBI populations. TBI severity also varied: 13 studies focused on severe TBI, 10 on moderate-to-severe TBI, and 17 included all TBI severities. Overall, 20 studies reported Injury Severity Scores (ISS; Supplementary Table 2), with a mean or median ISS > 16, reflecting serious injury. A total of 15 studies provided data on injury mechanisms, with blunt trauma predominating and penetrating injuries being rare.

### Mortality Outcomes in Individual Studies

Mortality was the primary outcome in 38 out of 40 studies (Table 2). Reporting of multiple mortality endpoints led to outcome counts exceeding the number of included studies, with in-hospital mortality most common (*n* = 31), while few reported 30-day (*n* = 3), 6-month (*n* = 8), ≥ 1-year (*n* = 2), prehospital (*n* = 1), or overall mortality (*n* = 1).

**Table 2:** Mortality (organized as female/male)

First Author	Year of publication	Mortality primary outcome?	Mortality percentage	Mortality (un)adjusted variables	*P*-value	Mortality OR/HR/RR	Significance	Conclusion mortality
Ahmed	2020	Yes: in-hospital	16.7%/36.9% X	**Crude** in-hospital mortality **Adjusted** in-hospital mortality for age, gender, race, SBP, HR, RR, ISS, smoking status, CKD status, CVA status, DM status, and HTN status	*P* < 0.001 *P* < 0.001	X ♂OR 1.963 (95% CI 1.688–2.283)	sig sig	**Females ↓ mortality** **Females ↓ mortality**
Albrecht	2016	Yes: in-hospital	16%/17% X X	**Crude** in-hospital mortality **isolated TBI** **Adjusted** in-hospital mortality **isolated TBI** for sex, age, race, AIS head score, GCS, cardiac arrhythmia, admission systolic blood pressure, and an indicator variable for transport to STC directly from scene **Adjusted** in-hospital mortality **non-isolated TBI** for sex, age, admission year, AIS head score, GCS, ISS, cardiac arrhythmia, admission systolic blood pressure, severity of other injuries, AIS face severity score, AIS spine severity score, AIS thorax severity score, AIS abdominal severity score, AIS upper extremity score, and an indicator variable for transport to STC directly from scene	*P* = 0.78 X X	♀OR 0.98 (95% CI 0.72–1.32) ♀OR 1.01 (95% CI 0.66–1.54) ♀OR 0.73 (95% CI 0.59–0.89)	ns ns sig	ND ND **Females ↓ mortality**
Beijer	2023	Yes: in-hospital, no: 30-day	34.2%/33.3% X 35.4%/34.3% X 21.3%/23.1% X 21.3%/22.8% X 37.6%/38.9% X 39.1%/40.7% X 31.6%/26.2% X 33.7%/28.7% X 12.5%/13.4% X 12.5%/13.4% X 34.6%/32.3% X 37.0%/35.9% X	**Crude** in-hospital mortality **Adjusted** in-hospital mortality for age, MOI, ISS, RTS < 4, GCS ≤ 8, SI, ASA ≥ III, ICP, and craniotomy **Crude** 30-day mortality **Adjusted** 30-day mortality for age, MOI, ISS, RTS < 4, GCS ≤ 8, SI, ASA ≥ III, ICP, and craniotomy **Crude** in-hospital mortality **16–44 y** **Adjusted** in-hospital mortality **16–44 y** for age, MOI, ISS, RTS < 4, GCS ≤ 8, SI, ASA ≥ III, ICP, and craniotomy **Crude** 30-day mortality **16–44 y** **Adjusted** 30-day mortality **16–44 y** for age, MOI, ISS, RTS < 4, GCS ≤ 8, SI, ASA ≥ III, ICP, and craniotomy **Crude** in-hospital mortality ≥ **45 y** **Adjusted** in-hospital mortality ≥ **45 y** for age, MOI, ISS, RTS < 4, GCS ≤ 8, SI, ASA ≥ III, ICP, and craniotomy **Crude** 30-day mortality ≥ **45 y** **Adjusted** 30-day mortality ≥ **45 y** for age, MOI, ISS, RTS < 4, GCS ≤ 8, SI, ASA ≥ III, ICP, and craniotomy **Crude** in-hospital mortality **isolated TBI** **Adjusted** in-hospital mortality **isolated TBI** for age, MOI, ISS, RTS < 4, GCS ≤ 8, SI, ASA ≥ III, ICP, and craniotomy **Crude** 30-day mortality **isolated TBI** **Adjusted** 30-day mortality **isolated TBI** for age, MOI, ISS, RTS < 4, GCS ≤ 8, SI, ASA ≥ III, ICP, and craniotomy **Crude** in-hospital mortality **isolated TBI 16–44 y** **Adjusted** in-hospital mortality **isolated TBI 16–44 y** for age, MOI, ISS, RTS < 4, GCS ≤ 8, SI, ASA ≥ III, ICP, and craniotomy **Crude** 30-day mortality **isolated TBI 16–44 y** **Adjusted** 30-day mortality **isolated TBI 16–44 y** for age, MOI, ISS, RTS < 4, GCS ≤ 8, SI, ASA ≥ III, ICP, and craniotomy **Crude** in-hospital mortality **isolated TBI ≥ 45 y** **Adjusted** in-hospital mortality **isolated TBI ≥ 45 y** for age, MOI, ISS, RTS < 4, GCS ≤ 8, SI, ASA ≥ III, ICP, and craniotomy **Crude** 30-day mortality **isolated TBI ≥ 45 y** **Adjusted** 30-day mortality **isolated TBI ≥ 45 y** for age, MOI, ISS, RTS < 4, GCS ≤ 8, SI, ASA ≥ III, ICP, and craniotomy	*P* = 0.704 *P* = 0.600 *P* = 0.677 *P* = 0.717 *P* = 0.691 *P* = 0.546 *P* = 0.734 *P* = 0.644 *P* = 0.681 *P* = 0.422 *P* = 0.607 *P* = 0.578 *P* = 0.095 *P* = 0.781 *P* = 0.132 *P* = 0.963 *P* = 0.883 *P* = 0.910 *P* = 0.883 *P* = 0.910 *P* = 0.548 *P* = 0.753 *P* = 0.788 *P* = 0.968	♀OR 1.044 (95% CI 0.836–1.304) ♀OR 1.074 (95% CI 0.822–1.405) ♀OR 1.048 (95% CI 0.841–1.306) ♀OR 1.051 (95% CI 0.803–1.375) ♀OR 0.900 (95% CI 0.535–1.513) ♀OR 0.818 (95% CI 0.426–1.571) ♀OR 0.914 (95% CI 0.543–1.537) ♀OR 0.858 (95% CI 0.449–1.640) ♀OR 0.948 (95% CI 0.737–1.221) ♀OR 1.132 (95% CI 0.836–1.534) ♀OR 0.936 (95% CI 0.729–1.203) ♀OR 1.090 (95% CI 0.804–1.478) ♀OR 1.303 (95% CI 0.954–1.779) ♀OR 1.056 (95% CI 0.719–1.550) ♀OR 1.264 (95% CI 0.932–1.715) ♀OR 0.991 (95% CI 0.674–1.457) ♀OR 0.925 (95% CI 0.329–2.605) ♀OR 1.083 (95% CI 0.273–4.302) ♀OR 0.925 (95% CI 0.329–2.605) ♀OR 1.083 (95% CI 0.273–4.302) ♀OR 1.109 (95% CI 0.791–1.556) ♀OR 1.067 (95% CI 0.712–1.600) ♀OR 1.047 (95% CI 0.751–1.459) ♀OR 0.992 (95% CI 0.660–1.489)	ns ns ns ns ns ns ns ns ns ns ns ns ns ns ns ns ns ns ns ns ns ns ns ns	ND ND ND ND ND ND ND ND ND ND ND ND ND ND ND ND ND ND ND ND ND ND ND ND
Berry	2009	Yes: in-hospital	9.4%/9.2% X 6.4%/6.1% 7.4%/9.6% 12.7%/16.1% X X X	**Crude** in-hospital mortality **Adjusted** in-hospital mortality for age groups (14–20, 21–45, 46–55, 56–65, 66–75, 76–89, and > 89 years), race (white, Black, Hispanic, Asian, Native American, and other), blunt/penetrating injury, systolic blood pressure < 90/ > 90, ISS > 16/ < 16, AIS > 3/ < 3 in the head, face, thorax, abdomen, spine, and any comorbidity (yes/no) **Crude** in-hospital mortality **14–44 y** **Crude** in-hospital mortality **45–54 y** **Crude** in-hospital mortality ≥ **55 y** **Adjusted** in-hospital mortality **14–44 y** for age groups (14–20, 21–45, 46–55, 56–65, 66–75, 76–89, and > 89 years), race (white, Black, Hispanic, Asian, Native American, and other), blunt/penetrating injury, systolic blood pressure < 90/ > 90, ISS > 16/ < 16, AIS > 3/ < 3 in the head, face, thorax, abdomen, spine, and any comorbidity (yes/no) **Adjusted** in-hospital mortality **45–54 y** for age groups (14–20, 21–45, 46–55, 56–65, 66–75, 76–89, and > 89 years), race (white, Black, Hispanic, Asian, Native American, and other), blunt/penetrating injury, systolic blood pressure < 90/ > 90, ISS > 16/ < 16, AIS > 3/ < 3 in the head, face, thorax, abdomen, spine, and any comorbidity (yes/no) **Adjusted** in-hospital mortality ≥ **55 y** for age groups (14–20, 21–45, 46–55, 56–65, 66–75, 76–89, and > 89 years), race (white, Black, Hispanic, Asian, Native American, and other), blunt/penetrating injury, systolic blood pressure < 90/ > 90, ISS > 16/ < 16, AIS > 3/ < 3 in the head, face, thorax, abdomen, spine, and any comorbidity (yes/no)	*P* = 0.2833 *P* < 0.0001 *P* = 0.4255 *P* = 0.0009 *P* < 0.0001 *P* = 0.0915 *P* = 0.0044 *P* < 0.0001	X ♀OR 0.82 (95% CI 0.77–0.88) X X X ♀OR 1.09 (95% CI 0.99–1.21) ♀OR 0.76 (95% CI 0.63–0.92) ♀OR 0.79 (95% CI 0.73–0.86)	ns sig ns sig sig ns sig sig	ND **Females ↓ mortality** ND **Females ↓ mortality** **Females ↓ mortality** ND **Females ↓ mortality** **Females ↓ mortality**
Chinese Head Trauma Study Collaborators	2021	Yes: in-hospital	7.22%/7.48% 20.72%/19.68%	**Crude** in-hospital mortality **Crude** in-hospital mortality **severe TBI**	*P* > 0.05 *P* > 0.05	X X	ns ns	ND ND
Coimbra	2003	Yes: in-hospital	3.8%/3.1% 3.0%/2.3% 1.1%/0.4% 0.9%/0% 5.0%/7.1% 5.6%/5.6% 33%/24% 27%/21%	**Crude** in-hospital mortality **all GCS** **Crude** in-hospital mortality **all GCS < 50 y** **Crude** in-hospital mortality **GCS 13–15** **Crude** in-hospital mortality **GCS 13–15 < 50 y** **Crude** in-hospital mortality **GCS 9–12** **Crude** in-hospital mortality **GCS 9–12 < 50 y** **Crude** in-hospital mortality **GCS < 9** **Crude** in-hospital mortality **GCS < 9 < 50 y**	*P* = 0.388 *P* > 0.05 *P* = 0.160 *P* > 0.05 *P* = 1.000 *P* > 0.05 *P* = 0.292 *P* > 0.05	X X X X X X X X	ns ns ns ns ns ns ns ns	ND ND ND ND ND ND ND ND
Colantonio	2008	Yes: after discharge ≥ 1 year	20.3%/18.9%	**Crude** after discharge mortality ≥ 1 year (TBI)	*P* = 0.100	X	ns	ND
Davis	2006	Yes: in-hospital	23.4%/22.8% 21.2%/20.3% 26.7%/31.2% 29.1%/34.4% X X X 20.7%/19.0% 22.1%/19.9% 21.2%/21.0% 20.3%/21.9% 16.2%/25.1% 25.0%/29.0% 30.3%/37.5% X X X X X X X	**Crude** in-hospital mortality **Crude** in-hospital mortality **15–49 y** **Crude** in-hospital mortality ≥ **50 y** **Crude** in-hospital mortality ≥ **60 y** **Adjusted** in-hospital mortality for age, mechanism of injury, Glasgow Coma Scale (GCS), hypotension (SBP < 90 mm Hg), head Abbreviated Injury Score (AIS), and Injury Severity Score (ISS) **Adjusted** in-hospital mortality **15–49 y** for age, mechanism of injury, Glasgow Coma Scale (GCS), hypotension (SBP < 90 mm Hg), head Abbreviated Injury Score (AIS), and Injury Severity Score (ISS) **Adjusted** in-hospital mortality ≥ **50 y** for age, mechanism of injury, Glasgow Coma Scale (GCS), hypotension (SBP < 90 mm Hg), head Abbreviated Injury Score (AIS), and Injury Severity Score (ISS) **Crude** in-hospital mortality **15–19 y** **Crude** in-hospital mortality **20–29 y** **Crude** in-hospital mortality **30–39 y** **Crude** in-hospital mortality **40–49 y** **Crude** in-hospital mortality **50–59 y** **Crude** in-hospital mortality **60–69 y** **Crude** in-hospital mortality ≥ **70 y** **Adjusted** in-hospital mortality **15–19 y** for age, mechanism of injury, pre-admission GCS score, the presence of pre-admission hypotension, head AIS, and ISS **Adjusted** in-hospital mortality **20–29 y** for age, mechanism of injury, pre-admission GCS score, the presence of pre-admission hypotension, head AIS, and ISS **Adjusted** in-hospital mortality **30–39 y** for age, mechanism of injury, pre-admission GCS score, the presence of pre-admission hypotension, head AIS, and ISS **Adjusted** in-hospital mortality **40–49 y** for age, mechanism of injury, pre-admission GCS score, the presence of pre-admission hypotension, head AIS, and ISS **Adjusted** in-hospital mortality **50–59 y** for age, mechanism of injury, pre-admission GCS score, the presence of pre-admission hypotension, head AIS, and ISS **Adjusted** in-hospital mortality **60–69 y** for age, mechanism of injury, pre-admission GCS score, the presence of pre-admission hypotension, head AIS, and ISS **Adjusted** in-hospital mortality ≥ **70 y** for age, mechanism of injury, pre-admission GCS score, the presence of pre-admission hypotension, head AIS, and ISS	*P* = ns *P* = ns *P* = sig *P* = NR *P* = ns *P* = ns *P* = sig *P* = ns *P* = ns *P* = ns *P* = ns *P* = sig *P* = ns *P* = sig *P* = ns *P* = ns *P* = ns *P* = ns *P* = sig *P* = ns *P* = sig	♀OR 1.03 (95% CI 0.94–1.14) ♀OR 1.06 (95% CI 0.93–1.19) ♀OR 0.80 (95% CI 0.69–0.93) X ♀OR 0.87 (95% CI 0.73–1.04) ♀OR 1.06 (95% CI 0.83–1.35) ♀OR 0.63 (95% CI 0.48–0.81) ♀OR 1.11 (95% CI 0.85–1.46) ♀OR 1.14 (95% CI 0.93–1.40) ♀OR 1.01 (95% CI 0.79–1.30) ♀OR 0.91 (95% CI 0.68–1.21) ♀OR 0.58 (95% CI 0.40–0.84) ♀OR 0.82 (95% CI 0.58–1.15) ♀OR 0.73 (95% CI 0.60–0.89) ♀OR 1.94 (95% CI 0.83–4.57) ♀OR 1.07 (95% CI 0.70–1.62) ♀OR 1.00 (95% CI 0.65–1.56) ♀OR 1.06 (95% CI 0.66–1.71) ♀OR 0.38 (95% CI 0.20–0.74) ♀OR 0.53 (95% CI 0.28–1.03) ♀OR 0.65 (95% CI 0.46–0.93)	ns ns sig NR ns ns sig ns ns ns ns sig ns sig ns ns ns ns sig ns sig	ND ND **Females ↓ mortality** NR ND ND **Females ↓ mortality** ND ND ND ND **Females ↓ mortality** ND **Females ↓ mortality** ND ND ND ND **Females ↓ mortality** ND **Females ↓ mortality**
de Guise	2014	Yes: in-hospital	10.1%/11.6% X	**Unadjusted** in-hospital mortality **Adjusted** in-hospital mortality for year, age, ISS, GCS, etiology, French speaking	*P* < 0.001 *P* = 0.02	X ♀OR 0.662 (95% CI 0.51–0.86)	sig sig	**Females ↓ mortality** **Females ↓ mortality**
Elkbuli	2020	Yes: in-hospital	4.5%/4.5%	**Crude** in-hospital mortality	*P* > 0.05	X	ns	ND
El-Menyar	2022	Yes: in-hospital	13.2%/12.5% 11.4%/11.7% 19.0%/21.1% X	**Crude** in-hospital mortality **Crude** in-hospital mortality **14–54 y** **Crude** in-hospital mortality ≥ **55 y** **Adjusted** in-hospital mortality for age, ISS, head AIS, comorbidities	*P* = 0.85 *P* = ns *P* = ns *P* = 0.640	♀OR 1.06 (95% CI 0.57–1.99) X X ♀OR 1.192 (95% CI 0.571–2.490)	ns ns ns ns	ND ND ND ND
Falk	2015	Yes: in-hospital	28%/30%	**Crude** in-hospital mortality	*P* = 0.28	X	ns	ND
Gan	2004	Yes: 6-month	X 70.4%/44.7%	**Crude** 6-month mortality **20–40 y** **Crude** 6-month mortality ≥ **64 y**	*P* = 0.232 *P* = 0.019	X X	ns sig	ND **Females ↑ mortality**
Gao	2017	Yes: 6-month	16.7%/25.6%	**Crude** 6-month mortality	*P* = ns	X	ns	ND
Gujral	2006	Yes: in-hospital, pre-hospital	5.6%/5.4% 12.9%/19.4% 6.0%/5.8% 49.2%/40.3% 10.2%/12.1% 88.5%/87.6% X X X X X X	**Crude** in-hospital mortality **Crude** pre-hospital mortality **Crude** in-hospital mortality **blunt** **Crude** in-hospital mortality **penetrating** **Crude** pre-hospital mortality **blunt** **Crude** pre-hospital mortality **penetrating** **Adjusted** in-hospital mortality for age, race, injury mechanism, CMSA **Adjusted** pre-hospital mortality for age, race, injury mechanism, CMSA **Adjusted** in-hospital mortality **blunt** for age, race, CMSA **Adjusted** in-hospital mortality **penetrating** for age, race, CMSA **Adjusted** pre-hospital mortality **blunt** for age, race, CMSA **Adjusted** pre-hospital mortality **penetrating** for age, race, CMSA	*P* = ns *P* = sig *P* = ns *P* = ns *P* < 0.05 *P* = ns *P* = sig *P* = sig *P* = sig *P* = ns *P* = sig *P* = ns	♂OR 1.05 (95% CI 0.92–1.19) ♂OR 1.62 (95% CI 1.50–1.76) X X X X ♂OR 1.19 (95% CI 1.05–1.37) ♂OR 1.21 (95% CI 1.10–1.34) ♂OR 1.20 (95% CI 1.04–1.39) ♂OR 0.90 (95% CI 0.51–1.59) ♂OR 1.20 (95% CI 1.09–1.34) ♂OR 0.99 (95% CI 0.73–1.35)	ns sig ns ns sig ns sig sig sig ns sig ns	ND **Females ↓ mortality** ND ND **Females ↓ mortality** ND **Females ↓ mortality** **Females ↓ mortality** **Females ↓ mortality** ND **Females ↓ mortality** ND
Herrera-Melero	2015	Yes: 6-month	36.8%/26% X	**Crude** 6-month mortality **Adjusted** 6-month mortality for age, ISS, GCS score, TCDB, pupillary alterations, hypotension, shock, anemia, hypothermia, intracranial hypertension, transfusion, MOF, and hyperglycemia	*P* = 0.039 *P* = ns	♀OR 1.65 (95% CI 1.02–2.67) X	sig ns	**Females ↑ mortality** ND
Hong	2020	Yes: in-hospital	7.9%/8.0% 3.7%/3.7% 6.1%/6.3% 9.1%/11.8% X X X	**Crude** in-hospital mortality **Crude** in-hospital mortality < **45 y** **Crude** in-hospital mortality **45–55 y** **Crude** in-hospital mortality > **55 y** **Adjusted** in-hospital mortality < **45 y** for ISS, GCS, race, smoking, CKD, diabetes, CAD, hypertension, obesity, respiratory disease, cirrhosis **Adjusted** in-hospital mortality **45–55 y** for ISS, GCS, race, smoking, CKD, diabetes, CAD, hypertension, obesity, respiratory disease, cirrhosis **Adjusted** in-hospital mortality > **55 y** for ISS, GCS, race, smoking, CKD, diabetes, CAD, hypertension, obesity, respiratory disease, cirrhosis	*P* = ns *P* = 0.964 *P* = 0.212 *P* < 0.001 *P* = 0.204 *P* = 0.001 *P* < 0.001	X X X X ♀OR 1.043 (95% CI 0.978–1.112) ♀OR 1.137 (95% CI 1.054–1.225) ♀OR 0.857 (95% CI 0.835–0.879)	ns ns ns sig ns sig sig	ND ND ND **Females ↓ mortality** ND **Females ↑ mortality** **Females ↓ mortality**
Hosomi	2021	Yes: in-hospital	12.01%/12.76% 4.9%/5.9% 14.0%/15.9% 14.37%/16.88% 2.83%/2.92% 2.47%/4.09% 6.34%/6.22% 4.95%/6.15% 7.88%/8.44% 10.31%/10.79% 12.34%/13.83% 13.54%/15.75% 15.71%/20.68% 17.17%/23.94% 15.15%/11.76% X X X X X X X X X X X	**Crude** in-hospital mortality **Crude** in-hospital mortality < **50 y** **Crude** in-hospital mortality ≥ **50 y** **Crude** in-hospital mortality ≥ **60 y** **Crude** in-hospital mortality** 0–9 y** **Crude** in-hospital mortality **10–19 y** **Crude** in-hospital mortality **20–29 y** **Crude** in-hospital mortality **30–39 y** **Crude** in-hospital mortality **40–49 y** **Crude** in-hospital mortality **50–59 y** **Crude** in-hospital mortality **60–69 y** **Crude** in-hospital mortality **70–79 y** **Crude** in-hospital mortality **80–89 y** **Crude** in-hospital mortality **90–99 y** **Crude** in-hospital mortality ≥ **100 y** **Adjusted** in-hospital mortality for age (10-year strata); year of TBI onset (2004–2006, 2007–2009, 2010–2012, 2013– 2015, 2016–2018); mechanism of trauma (motor vehicle driver, motor vehicle passenger, back seat passenger, motorcycle driver, motorcycle passenger, bicycle, pedestrian, other vehicle, fall from a great height, fall down the stairs, fall at ground level, other blunt injury, penetrating); alcohol drinking (no/yes); use of anticoagulant or antiplatelet drugs (no, yes); hypotension on admission to the emergency department (no, yes); Glasgow Coma Scale (GCS) group on arrival (3–15); maximum head AIS (3–5); type of TBI (diffuse brain injury, focal brain injury, uncategorized); site of TBI (1, 2, ≥ 3); whether an operation was indicated for TBI (no, yes), and post-TBI complications (none, neurological, non-neurological) **Adjusted** in-hospital mortality **0–9 y** for: see above **Adjusted** in-hospital mortality **10–19 y** for: see above **Adjusted** in-hospital mortality **20–29 y** for: see above **Adjusted** in-hospital mortality **30–39 y** for: see above **Adjusted** in-hospital mortality **40–49 y** for: see above **Adjusted** in-hospital mortality **50–59 y** for: see above **Adjusted** in-hospital mortality **60–69 y** for: see above **Adjusted** in-hospital mortality **70–79 y** for: see above **Adjusted** in-hospital mortality **80–89 y** for: see above **Adjusted** in-hospital mortality **90–99 y** for: see above	*P* = sig *P* = sig *P* = sig *P* = NR *P* = ns *P* = sig *P* = ns *P* = ns *P* = ns *P* = ns *P* = ns *P* = sig *P* = sig *P* = sig *P* = ns *P* = sig *P* = ns *P* = sig *P* = ns *P* = ns *P* = ns *P* = ns *P* = sig *P* = sig *P* = sig *P* = sig	♂OR 1.07 (95% CI 1.01–1.13) X X X ♂OR 1.03 (95% CI 0.60–1.80) ♂OR 1.68 (95% CI 1.04–2.72) ♂OR 0.98 (95% CI 0.69–1.39) ♂OR 1.26 (95% CI 0.84–1.88) ♂OR 1.08 (95% CI 0.81–1.43) ♂OR 1.05 (95% CI 0.85–1.30) ♂OR 1.14 (95% CI 0.99–1.31) ♂OR 1.19 (95% CI 1.07–1.33) ♂OR 1.40 (95% CI 1.26–1.55) ♂OR 1.52 (95% CI 1.22–1.89) ♂OR 0.75 (95% CI 0.13–4.32) ♂OR 1.32 (95% CI 1.22–1.42) ♂OR 0.94 (95% CI 0.43–2.02) ♂OR 1.97 (95% CI 1.08–3.61) ♂OR 1.16 (95% CI 0.72–1.87) ♂OR 1.14 (95% CI 0.67–1.94) ♂OR 1.08 (95% CI 0.74–1.58) ♂OR 1.14 (95% CI 0.86–1.51) ♂OR 1.24 (95% CI 1.02–1.50) ♂OR 1.20 (95% CI 1.03–1.40) ♂OR 1.50 (95% CI 1.31–1.73) ♂OR 1.72 (95% CI 1.28–2.32)	sig sig sig NR ns sig ns ns ns ns ns sig sig sig ns sig ns sig ns ns ns ns sig sig sig sig	**Females ↓ mortality** **Females ↓ mortality** **Females ↓ mortality** NR ND **Females ↓ mortality** ND ND ND ND ND **Females ↓ mortality** **Females ↓ mortality** **Females ↓ mortality** ND **Females ↓ mortality** ND **Females ↓ mortality** ND ND ND ND **Females ↓ mortality** **Females ↓ mortality** **Females ↓ mortality** **Females ↓ mortality**
Jung	2024	Yes: 6-month, no: 1-month, ED	17.3%/18.9% 10.6%/13.7% 8.0%/11.3%	**Crude** 6-month mortality **Crude** 1-month mortality **Crude** ED mortality	*P* = 0.124 *P* = ns *P* = 0.059	X X X	ns ns ns	ND ND ND
Kadar	2019	Yes: in-hospital	17.3%/22.1%	**Crude** in-hospital mortality	*P* = sig	♂OR 1.36 (95% CI 1.10–1.68)	sig	**Females ↓ mortality**
Khan	2019	Yes: in-hospital	6.7%/9.9%	**Crude** in-hospital mortality	*P* = 0.159	X	ns	ND
Kirkness	2004	Yes: in-hospital, 6-month	15%/13% 18.2%/16.9%	**Crude** in-hospital mortality **Crude** 6-month mortality	*P* = ns *P* = ns	X X	ns ns	ND ND
Klauber	1981	Yes: 1-year	2.6%/3.0% 4.4%/3.5% 1.9%/3.2% 5.5%/5.7% 4.3%/3.2% 2.0%/0.5% 1.5%/3.2% 4.6%/2.0% 1.6%/3.1% 5.0%/4.6% 4.1%/4.2% 5.9%/5.6% 7.5%/7.1% 5.6%/12.1%	**Crude** 1-year mortality **all injuries** **Crude** 1-year mortality **selected hospitals** **Crude** 1-year mortality **all injuries < 40 y** **Crude** 1-year mortality **all injuries ≥ 40 y** **Crude** 1-year mortality** all injuries 0–9 y** **Crude** 1-year mortality** selected hospitals 0–9 y** **Crude** 1-year mortality** all injuries 10–19 y** **Crude** 1-year mortality** selected hospitals 10–19 y** **Crude** 1-year mortality** all injuries 20–39 y** **Crude** 1-year mortality** selected hospitals 20–39 y** **Crude** 1-year mortality** all injuries 40–59 y** **Crude** 1-year mortality** selected hospitals 40–59 y** **Crude** 1-year mortality **all injuries ≥ 60 y** **Crude** 1-year mortality **selected hospitals ≥ 60 y**	*P* = ns *P* = ns *P* = sig *P* = ns *P* = ns *P* = ns *P* = ns *P* = ns *P* = ns *P* = ns *P* = ns *P* = ns *P* = ns *P* = ns	X X X X X X X X X X X X X X	ns ns sig ns ns ns ns ns ns ns ns ns ns ns	ND ND **Females ↓ mortality** ND ND ND ND ND ND ND ND ND ND ND
Kokkinou	2020	Yes: in-hospital	3%/9%	**Crude** in-hospital mortality	*P* = ns	X	ns	ND
Kraus	2000	Yes: overall, < 1-h, > 1 to < 6-h, > 6-h, post discharge until 18 months	31.6%/24.7% 3.5%/2.9% 6.6%/6.2% 20.3%/15.3% 11.4%/5.3% X X 18.4%/26.0% 23.4%/20.0% 51.1%/28.4%	**Crude** overall mortality **Crude** mortality < 1 h **Crude** mortality > 1 to < 6 h **Crude** mortality > 6 h to prior to discharge **Crude** mortality post discharge until 18 months **Unadjusted** overall mortality **Adjusted** overall mortality for age, admission GCS, blunt/penetrating injury, multiple trauma **Crude** overall mortality **16–29 y** **Crude** overall mortality **30–49 y** **Crude** overall mortality ≥ **50 y**	X X X X X X X X X X	♀OR 1.28 (95% CI 0.98–1.69) ♀OR 1.20 (95% CI 0.46–3.16) ♀OR 1.06 (95% CI 0.53–2.13) ♀OR 1.33 (95% CI 0.90–1.97) ♀OR 2.14 (95% CI 0.79–5.77) ♀OR 1.39 (95% CI 0.93–2.07) ♀OR 1.75 (95% CI 1.09–2.82) X X		
X	ns ns ns ns ns ns **sig** ns ns ns	ND ND ND ND ND ND **Females ↑ mortality** ND ND ND						
Leitgeb	2011	Yes: in-hospital	39.6%/32.5% X	**Crude** in-hospital mortality **Adjusted** in-hospital mortality for age, pupillary reactivity, ISS, head AIS, GCS score, Rotterdam CT score, injury mechanism, presence of subdural hematoma	*P* = 0.16 X	X X	ns ns	ND ND
Ley	2013	Yes: in-hospital	6.6%/7.0% 5.4%/5.0% 8.2%/8.8% X X	**Crude** in-hospital mortality **Crude** in-hospital mortality **0–12 y** **Crude** in-hospital mortality **13–18 y** **Adjusted** in-hospital mortality **0–12 y** for ISS 16–24, ISS ≥ 25, GCS ≤ 8, SBP < 90 mm Hg **Adjusted** in-hospital mortality **13–18 y** for ISS 16–24, ISS ≥ 25, GCS ≤ 8, SBP < 90 mm Hg	*P* = ns *P* = 0.43 *P* = 0.38 *P* = 0.63 *P* = 0.007	X X X ♀OR 1.05 (95% CI 0.85–1.30) ♀OR 0.78 (95% CI 0.65–0.93)	ns ns ns ns sig	ND ND ND ND **Females ↓ mortality**
Mair	2022	Yes: in-hospital, trauma room, 6-h, 12-h, 24-h, 48-h, 7-day, 30-day	29.2%/30.8% 1.3%/1.2% 4.1%/4.4% 10.6%/10.1% 15.8%/14.7% 19.0%/17.4% 23.8%/23.4% 28.6%/30.0% 13.4%/13.1% 1.4%/1.4% 2.5%/2.8% 5.0%/4.5% 6.9%/6.0% 8.3%/8.1% 11.3%/10.6% 13.3%/12.9% 31.8%/33.8% 1.3%/1.1% 4.4%/4.6% 11.5%/11.1% 17.3%/16.1% 20.7%/19.0% 25.9%/25.5% 31.1%/32.9%	**Crude** in-hospital mortality **Crude** trauma room mortality **Crude** 6-h mortality **Crude** 12-h mortality **Crude** 24-h mortality **Crude** 48-h mortality **Crude** 7-day mortality **Crude** 30-day mortality **Crude** in-hospital mortality ≤ **45 y** **Crude** trauma room mortality ≤ **45 y** **Crude** 6-h mortality ≤ **45 y** **Crude** 12-h mortality ≤ **45 y** **Crude** 24-h mortality ≤ **45 y** **Crude** 48-h mortality ≤ **45 y** **Crude** 7-day mortality ≤ **45 y** **Crude** 30-day mortality ≤ **45 y** **Crude** in-hospital mortality > **45 y** **Crude** trauma room mortality > **45 y** **Crude** 6-h mortality > **45 y** **Crude** 12-h mortality > **45 y** **Crude** 24-h mortality > **45 y** **Crude** 48-h mortality > **45 y** **Crude** 7-day mortality > **45 y** **Crude** 30-day mortality > **45 y**	*P* = 0.005 *P* = 0.406 *P* = 0.364 *P* = 0.304 *P* = 0.014 *P* = 0.002 *P* = 0.415 *P* = 0.014 *P* = 0.839 *P* = 1.010 *P* = 0.671 *P* = 0.571 *P* = 0.328 *P* = 0.851 *P* = 0.546 *P* = 0.760 *P* = 0.003 *P* = 0.346 *P* = 0.454 *P* = 0.373 *P* = 0.022 *P* = 0.002 *P* = 0.519 *P* = 0.007	X X X X X X X X X X X X X X X X X X X X X X X X	sig ns ns ns sig sig ns sig ns ns ns ns ns ns ns ns sig ns ns ns sig sig ns sig	**Females ↓ mortality** ND ND ND **Females ↑ mortality** **Females ↑ mortality** ND **Females ↓ mortality** ND ND ND ND ND ND ND ND **Females ↓ mortality** ND ND ND **Females ↑ mortality** **Females ↑ mortality** ND **Females ↓ mortality**
Martins	2009	Yes: in-hospital	39.3%/32.2%	**Crude** in-hospital mortality	*P* = 0.13	♀OR 1.36 (95% CI 0.91–2.05)	ns	ND
Mikolić	2021	Yes: in-hospital	6.4%/8.5% 2.2%/2.0% 20.9%/22.0% X X	**Crude** in-hospital mortality **Crude** in-hospital mortality **mild TBI** **Crude** in-hospital mortality** moderate–severe TBI** **Adjusted** in-hospital mortality for age, baseline Glasgow Coma Scale score, pupillary reactivity, hypotension and hypoxia before arrival/at admission, Marshall Classification, traumatic subarachnoid hemorrhage (tSAH), epidural hematoma, Injury Severity Score (ISS), pre-injury medical situation (ASA PS Classification), pre-injury psychiatric disorder, and cause of injury **Adjusted** 6-month mortality for age, baseline Glasgow Coma Scale score, pupillary reactivity, hypotension and hypoxia before arrival/at admission, Marshall Classification, traumatic subarachnoid hemorrhage (tSAH), epidural hematoma, Injury Severity Score (ISS), pre-injury medical situation (ASA PS Classification), pre-injury psychiatric disorder, and cause of injury	*P* = sig *P* = 0.863 *P* = 0.717 *P* = 0.2 *P* = 0.08	X X X ♀OR 0.8 (95% CI 0.5–1.2) ♀OR 0.7 (95% CI 0.5–1.0)	sig ns ns ns ns	**Females ↓ mortality** ND ND ND ND
Morrison	2004	Yes: in-hospital	6.1%/5.3% 15%/13.7% X 0.2%/0% 0.9%/0.9% 20%/19% 61%/53% 0%/0.1% 0.4%/0.7% 10%/11% 54%/53% 0%/0% 0%/0.1% 8%/8% 51%/42%	**Crude** in-hospital mortality **Crude** in-hospital mortality** severe** **Adjusted** in-hospital mortality for age, ISS, and MVC **Crude** in-hospital mortality **0–7 y mild** **Crude** in-hospital mortality **0–7 y moderate** **Crude** in-hospital mortality **0–7 y severe** **Crude** in-hospital mortality **0–7 y life-threatening** **Crude** in-hospital mortality **8–12 y mild** **Crude** in-hospital mortality **8–12 y moderate** **Crude** in-hospital mortality **8–12 y severe** **Crude** in-hospital mortality **8–12 y life-threatening** **Crude** in-hospital mortality **13–19 y mild** **Crude** in-hospital mortality **13–19 y moderate** **Crude** in-hospital mortality **13–19 y severe** **Crude** in-hospital mortality **13–19 y life-threatening**	*P* = 0.03 *P* = NR *P* = 0.22 *P* = NR *P* = ns *P* = ns *P* = ns *P* = NR *P* = ns *P* = ns *P* = ns *P* = NR *P* = NR *P* = ns *P* = ns	X X ♂OR 0.9 (95% CI 0.76–1.07) X X X X X X X X X X X X	sig NR ns NR ns ns ns NR ns ns ns NR NR ns ns	**Females ↑ mortality** NR ND NR ND ND ND NR ND ND ND NR NR ND ND
Munivenkatappa	2016	Yes: in-hospital	3.4%/1.6% 1.3%/0.5% 3.6%/1.6% 7.1%/4.8%	**Crude** in-hospital mortality **Crude** in-hospital mortality ≤ **18 y** **Crude** in-hospital mortality **18–60 y** **Crude** in-hospital mortality ≥ **61 y**	*P* = 0.048 *P* = ns *P* = ns *P* = ns	X X X X	sig ns ns ns	**Females ↑ mortality** ND ND ND
Ng	2006	Yes: 6-month	42.9%/33.7% X X X X X	**Crude** 6-month mortality **Adjusted** 6-month mortality for age, multiple injuries, post-resuscitation pupil abnormalities, and GCS **Crude** 6-month mortality ≤ **60 y** **Adjusted** 6-month mortality ≤ **60 y** for age, multiple injuries, post-resuscitation pupil abnormalities, and GCS **Crude** 6-month mortality > **60 y** **Adjusted** 6-month mortality > **60 y** for age, multiple injuries, post-resuscitation pupil abnormalities, and GCS	*P* = 0.042 *P* = 0.396 *P* = 0.807 *P* = 0.407 *P* = 0.490 *P* = 0.587	♀OR 1.48 (95% CI 1.02–2.14) ♀OR 0.79 (95% CI 0.46–1.36) ♀OR 0.94 (95% CI 0.54–1.61) ♀OR 0.74 (95% CI 0.36–1.52) ♀OR 1.25 (95% CI 0.67–2.33) ♀OR 0.80 (95% CI 0.36–1.79)	sig ns ns ns ns ns	**Females ↑ mortality** ND ND ND ND ND
O’Reilly	2023	No: in-hospital	16.0%/14.5%	**Crude** in-hospital mortality	*P* = sig	X	sig	**Females ↑ mortality**
Ottochian	2009	Yes: in-hospital	41.9%/29.5% 22.7%/31.0% 30.1%/24.7% 40.0%/28.5% 54.9%/38.3% X X X X X	**Crude** in-hospital mortality **Crude** in-hospital mortality < **14 y** **Crude** in-hospital mortality **14–44 y** **Crude** in-hospital mortality **45–54 y** **Crude** in-hospital mortality ≥ **55 y** **Adjusted** in-hospital mortality for age, sex, admission systolic blood pressure, Glasgow Coma Scale score on admission, injury severity score, ethnicity, and toxicology **Adjusted** in-hospital mortality < **14 y** for age, sex, admission systolic blood pressure, Glasgow Coma Scale score on admission, injury severity score, ethnicity, and toxicology **Adjusted** in-hospital mortality **14–44 y** for age, sex, admission systolic blood pressure, Glasgow Coma Scale score on admission, injury severity score, ethnicity, and toxicology **Adjusted** in-hospital mortality **45–54 y** for age, sex, admission systolic blood pressure, Glasgow Coma Scale score on admission, injury severity score, ethnicity, and toxicology **Adjusted** in-hospital mortality ≥ **55 y** for age, sex, admission systolic blood pressure, Glasgow Coma Scale score on admission, injury severity score, ethnicity, and toxicology	*P* < 0.0001 *P* = 0.319 *P* = 0.211 *P* = 0.096 *P* < 0.0001 *P* = 0.02 *P* = 0.39 *P* = 0.29 *P* = 0.41 *P* = 0.015	X X X X X ♀OR 1.4 (95% CI 1.1–1.9) ♀OR 0.6 (95% CI 0.2–2.0) ♀OR 1.3 (95% CI 0.8–2.3) ♀OR 1.4 (95% CI 0.6–3.2) ♀OR 1.7 (95% CI 1.1–2.6)	sig ns ns ns sig sig ns ns ns sig	**Females ↑ mortality** ND ND ND **Females ↑ mortality** **Females ↑ mortality** ND ND ND **Females ↑ mortality**
Rappold	2002	Yes: in-hospital	2.7%/2.9% 2.1%/1.7% 34.7%/26.5%	**Crude** in-hospital mortality **Crude** in-hospital mortality **TBI and SBP > 90** **Crude** in-hospital mortality **TBI and SBP < 90**	X X X	X X X	ns ns ns	ND ND ND
Saatian	2018	Yes: in-hospital	3.0%/5.7% 1.9%/3.8% 7.1%/15.3% 1.1%/1.5% 1.9%/3.3% 1.7%/4.3% 2.9%/4.7% 3.3%/7.0% 4.5%/10.1% 6.3%/12.5% 12.6%/22.1% 8.1%/29.5% 12.5%/28.6% 0%/100% X	**Crude** in-hospital mortality **Crude** in-hospital mortality ≤ **50 y** **Crude** in-hospital mortality > **50 y** **Crude** in-hospital mortality **0–10 y** **Crude** in-hospital mortality **11–20 y** **Crude** in-hospital mortality **21–30 y** **Crude** in-hospital mortality **31–40 y** **Crude** in-hospital mortality **41–50 y** **Crude** in-hospital mortality **51–60 y** **Crude** in-hospital mortality **61–70 y** **Crude** in-hospital mortality **71–80 y** **Crude** in-hospital mortality **81–90 y** **Crude** in-hospital mortality **91–100 y** **Crude** in-hospital mortality **101–110 y** **Adjusted** in-hospital mortality for age, surgery, LOH, and trauma type	X X X X X X X X X X X X X X *P* ≤ 0.001	X X X X X X X X X X X X X X ♂OR 1.52 (95% CI 1.18–1.96)	X X X X X X X X X X X X X X sig	NR NR NR NR NR NR NR NR NR NR NR NR NR NR **Females ↓ mortality**
Shibahashi	2017	Yes: in-hospital	3.34%/4.1% X	**Crude** in-hospital mortality **Adjusted** in-hospital mortality for age; year of admittance; GCS score on arrival at the hospital; comorbidities; hypotension (< 90 mm Hg systolic) on arrival; RTS; ISS; whether head CT was performed for initial surveying; and the nature of the head injury	*P* = NR *P* < 0.001	X ♂OR 1.50 (95% CI 1.27–1.77)	NR sig	NR **Females ↓ mortality**
Svedung Wettervik	2022	No: 6-month	11%/11% X	**Crude** 6-month mortality **Adjusted** 6-month mortality for age, GCS, bilateral unreactive pupils	*P* = 1.00 *P* = 0.64	X ♀OR 1.21 (95% CI 0.543–2.70)	ns ns	ND ND
Yeung	2011	Yes: in-hospital	12.2%/10.1% 3.8%/4.9% X 12.4/12.9% 5.2%/8.9% X	**Crude** in-hospital mortality **(Australia)** **Crude** in-hospital mortality** (Australia) isolated TBI** **Adjusted** in-hospital mortality **(Australia)** for isolated head injury and multiple trauma, causes of injury, comorbidity status, transferred patients, trauma call, length of stay, systolic blood pressure, respiratory rate, Glasgow Coma Scale score, intensive care unit admission, head injury-related operation, and Injury Severity Score **Crude** in-hospital mortality **(China)** **Crude** in-hospital mortality **(China) isolated TBI** **Adjusted** in-hospital mortality **(China)** for isolated head injury and multiple trauma, causes of injury, comorbidity status, transferred patients, trauma call, length of stay, systolic blood pressure, respiratory rate, Glasgow Coma Scale score, intensive care unit admission, head injury-related operation, and Injury Severity Score	*P* = 0.20 *P* = 0.68 *P* = 0.36 *P* = 0.88 *P* = 0.30 *P* = 0.20	♂OR 0.81 (95% CI 0.59–1.11) ♂OR 1.29 (95% CI 0.37–4.48) ♂OR 0.72 (95% CI 0.35–1.45) ♂OR 1.04 (95% CI 0.62–1.75) ♂OR 1.784 (95% CI 0.592–5.364) ♂OR 2.07 (95% CI 0.67–6.39)	ns ns ns ns ns ns	ND ND ND ND ND ND

*AIS* Abbreviated Injury Scale score; *ASA* American Society of Anesthesiologists classification; *CI* confidence interval; *CKD* chronic kidney disease; *CT* computed tomography; *CVA* cerebro vasculair accident; *DM* diabetes mellitus; *ED* emergency department; *HR* heart rate; *HTN* hypertension; *ICP* intracranial pressure; *ISS* Injury Severity Score; *LOH* length of hospitalization; *MOI* mechanism of injury; *ND* no difference; *NR* not reported; *ns* not statistically significant; *RR* respiratory rate; *RTS* Revised Trauma Score; *SBP* systolic blood pressure; *STC* R Adams Cowley Shock Trauma Center; *y* years of age; *sig* significant

#### Overall Mortality: Crude and Adjusted

**Fig. 2:**
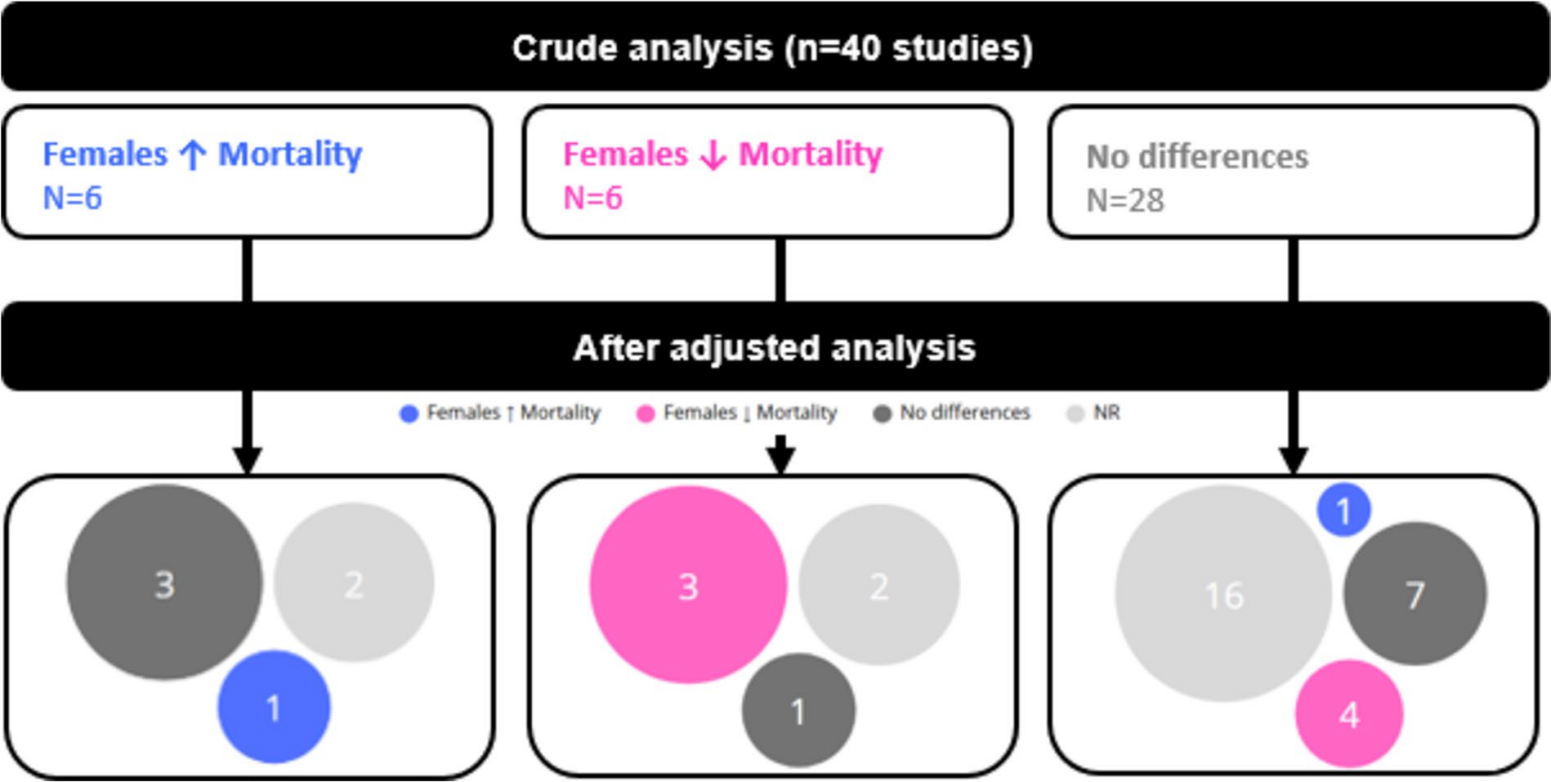
Mortality flow diagram flow diagram summarizing crude and adjusted results on mortality of all included studies NR, not reported

Of the 40 studies, 6 [35, 43, 52, 54, 62, 64] reported lower crude mortality for female compared with male patients, 28 found no crude difference, and 6 [50, 65–69] reported higher crude mortality in female individuals. In the six studies reporting lower crude female mortality, this association persisted after adjustment in three studies [35, 43, 52] and disappeared in one study [64]. In addition, two studies [54, 62] did not report adjusted analyses. Among studies reporting higher crude female mortality, one [69] remained significant after adjustment, three [50, 65, 67] became nonsignificant, and two [66, 68] did not adjust. Of the 28 studies reporting no crude differences, 4 [38, 49, 71, 72] showed lower adjusted mortality in female patients, and 1 [59] reported higher adjusted female mortality (Fig. 2).

#### Subgroup: Age

Many studies stratified outcome by age (Table 2 and Supplementary Tables 1 and 2). Although different cutoffs for age were used across studies, we classified studies into a younger age subgroup < 50 years to approximate premenopausal and ≥ 60 years to approximate postmenopausal groups. In the younger subgroup, three studies [38, 52, 57] reported lower mortality for female individuals in crude analyses; however, this association was no longer present after adjusting for covariates. Among studies performed in older patients, three studies [35, 42, 52] reported lower unadjusted mortality among female patients, while one study [47] found higher female mortality. After adjustment, two studies still reported lower mortality in post-menopausal female patients.

Four studies [61, 65, 66, 69] reported analyses restricted to pediatric subgroups. Three of these studies, spanning children and adolescents, found no sex-based difference in crude (and when reported, adjusted) mortality [65, 66, 69]. In contrast, one study observed lower adjusted mortality among adolescent female patients (13–18 years). [61]

#### Subgroup: TBI Severity

Overall, 10 studies focused on patients with moderate-to-severe TBI, 13 specifically focused on severe TBI, and 17 considered all TBI severities. Among severe TBI studies, crude analyses more frequently suggested higher mortality in female patients [50, 67, 69] and one suggested [62] lower mortality. After adjustment, only one study [69] continued to show higher mortality in female individuals. For moderate-to-severe TBI, studies reported both higher and lower mortality in female patients, as well as no difference (Table 2 and Supplementary Tables 1 and 2).

#### Subgroup: Isolated TBI

Among 11 studies reporting on isolated TBI (Table 2 and Supplementary Tables 1 and 2), 2 [52, 62] demonstrated lower crude mortality in female patients, while 1 [69] reported higher mortality in female compared with male patients. Adjusted analyses similarly showed both lower [38, 52, 72] or higher [69] mortality in female individuals.

#### Subgroup: Mechanism of Injury

Six studies specifically reported outcomes for blunt injuries [49, 51, 61, 65, 69, 70], while the other studies did not specify between blunt and penetrating mechanisms (Table 2 and Supplementary Tables 1 and 2). Crude analyses in two studies [65, 69] suggest higher mortality in female patients with blunt TBI; however, adjustment attenuated this association in one study. Another study [49] reported lower female mortality after blunt TBI in crude and adjusted analysis, whereas no difference was observed between sexes after penetrating injuries.

### Pooled Results: Meta-Analysis and Sensitivity Analyses

**Fig. 3:**
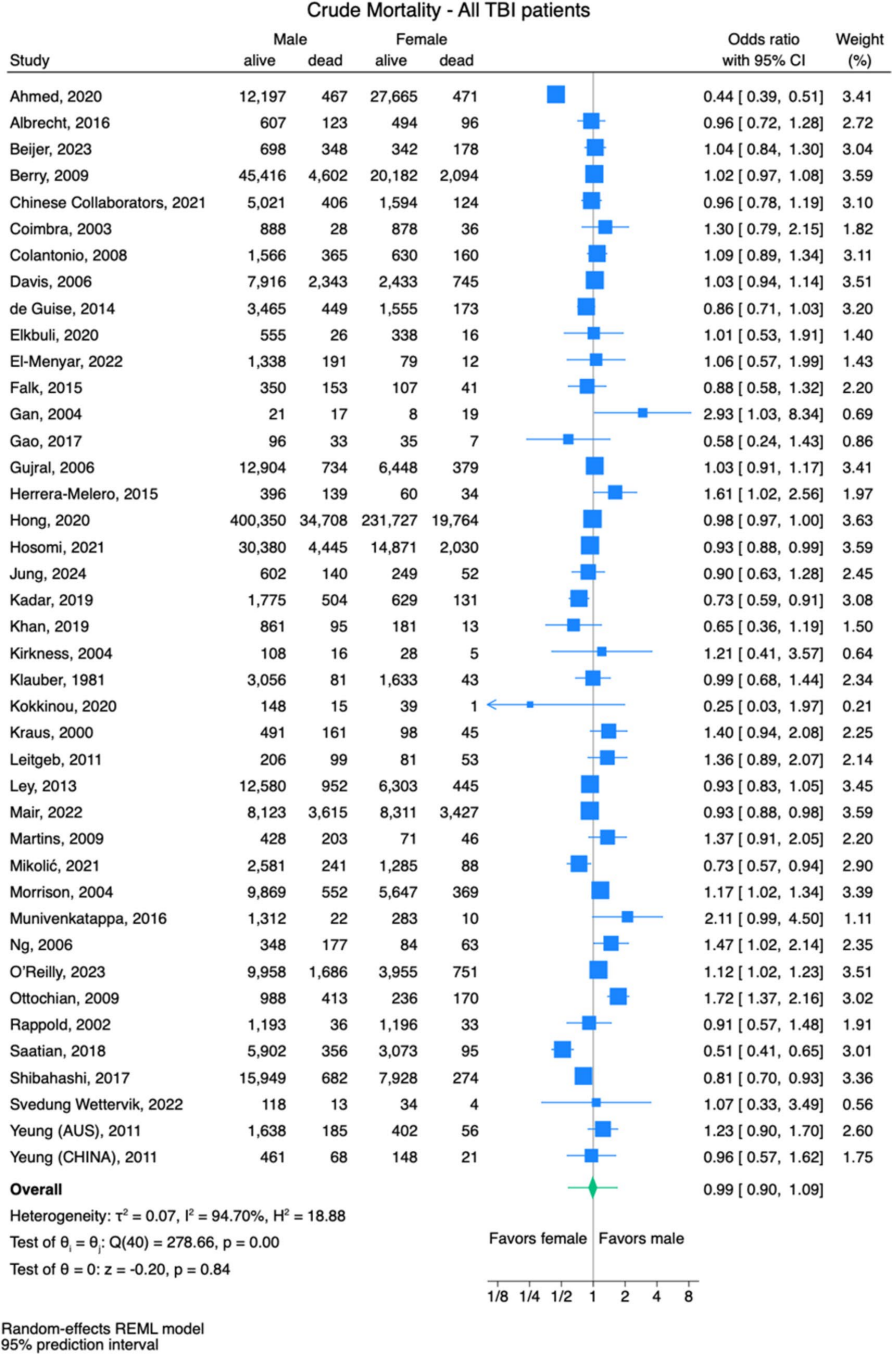
Forest plot Forest plot summarizing the individual studies and pooled results of the meta-analysis

All 40 studies were included in the meta-analysis (Supplementary Table 1). Egger’s test provided no evidence for small-study bias (*p* = 0.24; Supplementary Fig. 1). Overall, no significant association between sex and TBI mortality was observed (OR 0.99, 95% CI 0.90–1.09, 95% PI 0.58–1.68, *p* = 0.84; Fig. 3). When stratified by study quality, studies with poor and fair quality showed no association (OR 1.00, 95% CI 0.87–1.15, 95% PI 0.52–1.94, *p* = 0.96 and OR 1.20, 95% CI 0.90–1.61, 95% PI 0.44–3.26, *p* = 0.21 respectively), while good-quality studies showed slightly lower mortality in female patients (OR 0.95, 95% CI 0.91–0.99, 95% PI 0.86–1.06, *p* = 0.02; Supplementary Fig. 2). However, the subgroup difference test was not significant (*p* = 0.24). Moreover, leave-one-out sensitivity analysis indicated that this result was driven by two studies, [52, 62] and removing either rendered the association nonsignificant. A sensitivity analysis restricted to in-hospital mortality showed results comparable to the analysis including all studies (OR 0.95, 95% CI 0.86–1.06, 95% PI 0.56–1.64, *p* = 0.39; Supplementary Fig. 3).

Subgroup analysis for TBI type (isolated TBI vs. mixed TBI or not specified) showed no association between sex and mortality in either group (OR 1.02, 95% CI 0.90–1.16, 95% PI 0.67–1.57, *p* = 0.72 and OR 0.98, 95% CI 0.87–1.11, 95% PI 0.54–1.78, *p* = 0.74 respectively; Supplementary Figs. 4and 5). For TBI severity, pooled estimates showed no sex-related differences in moderate-to-severe TBI (OR 1.00, 95% CI 0.93–1.08, 95% PI 0.82–1.23, *p* = 0.93) and a modest association with higher mortality in female patients in severe TBI (OR 1.18, 95% CI 1.03–1.36, 95% PI 0.76–1.83, *p* = 0.02; Supplementary Figs. 6 and 7). Notably, this finding was largely driven by a single study [69]; excluding it in leave-out-one analyses nullified the significance. Stratified analyses by age group (pediatric, < 50 years, ≥ 60 years) showed no significant sex differences in mortality (OR 1.02, 95% CI 0.83–1.26, 95% PI 0.48–2.19, *p* = 0.86; OR 0.79, 95% CI 0.58–1.07, 95% PI 0.23–2.71, *p* = 0.12; OR 0.84, 95% CI 0.56–1.27, 95% PI 0.22–3.22, *p* = 0.41; Supplementary Fig. 8). Subgroup analysis based on study period (before vs. after 2000s), demonstrated a modest association with higher female mortality in studies conducted before the 2000s (OR 1.08, 95% CI 1.02–1.14, *p* = 0.01; Supplementary Fig. 9); however, this association was not significant when compared with studies conducted after the 2000s in the test for subgroup differences (*p* = 0.08).

Substantial heterogeneity was observed overall (*I*^2^ 94.7%). Residual heterogeneity was lower, but still present, in some stratified analyses (e.g., good-quality studies *I*^2^ 58.6%, severe TBI subgroup *I*^2^ 67.6%, pediatric subgroup *I*^2^ 62.6%, before 2000s *I*^2^ 0.01%), whereas heterogeneity remained high in others (e.g., age subgroups; Supplementary Figs. 2–9).

In a random-effects meta-regression including 23 studies, the difference in mean age between female and male individuals was not associated with the crude sex–mortality log(OR) (*β* = 0.002, 95% CI − 0.018 to 0.022; *p* = 0.85). Furthermore, age differences explained none of the between-study variance (*R*^2^ 0.00%), with substantial residual heterogeneity remaining (*I*^2^ 84.26%).

## Discussion

In this systematic review and meta-analysis, we found no significant overall association between sex and mortality following TBI. Importantly, in the presence of substantial between-study heterogeneity, and under a random-effects framework, this near-null average effect should not be interpreted as evidence that sex has no relationship with mortality. Instead, it suggests that the sex–mortality relationship varies across study contexts depending on setting, patient selection, injury characteristics, and other factors. The wide 95% prediction intervals are compatible with a scenario in which female sex is associated with higher mortality in some settings and lower mortality in others, yielding a pooled effect close to unity. This lack of a consistent overall effect persisted across most subgroups, including isolated versus mixed TBI, age groups, and moderate-to-severe TBI. Initial pooling suggested potential associations in specific subsets, such as lower mortality for female patients in good-quality studies or higher mortality in severe TBI and studies conducted before the 2000s; however, sensitivity analyses revealed that significant findings were generally driven by single studies, cautioning against broad generalizations and reinforcing that specific study and patient characteristics, rather than biological sex alone, likely drive the observed outcomes.

Five previous reviews examining sex and outcomes in TBI populations, including four meta-analyses and one scoping review, were identified [8–12]. While this recent surge in publication, with three reviews published in 2024 alone, highlights the importance of the topic, current literature remains fragmented and limited in scope. Specifically, previous efforts have been restricted by narrow inclusion criteria or a focus on outcomes other than mortality. Early work is now outdated and included only a single study on mortality [8], while more recent reviews did not quantitatively meta-analyze mortality [12], focused on nonmortality outcomes or specific severity subgroups, and were limited by small study pools (*n* = 6 and *n* = 11) [9, 10], or restricted their scope to geriatric patients [11]. Consequently, a comprehensive synthesis of mortality data across the full spectrum of TBI severity and age groups has been lacking. Our study addresses this gap, providing the most comprehensive analysis to date by synthesizing data from 40 studies and over 1 million patients.

A key methodological consideration in sex-related research is the interpretation of crude versus adjusted analyses. Some of the included studies reported only crude outcomes, whereas others adjusted for clinical factors such as injury severity and mechanism of injury. Because these variables may plausibly lie on causal pathways downstream of sex, statistical adjustment can attenuate estimates of the total sex–mortality association and instead target a different estimand, namely, a conditional or “direct/independent” association. Inconsistent reporting of adjusted effect estimates and heterogeneous covariate sets across studies further limit comparability and interpretability. Accordingly, our synthesis focused on crude sex-stratified mortality to summarize the total observed association. However, unadjusted estimates remain vulnerable to confounding by nonmediating factors, particularly age, and female patients were often older than male patients. Our exploratory meta-regression found no association between sex-specific age imbalance and the crude sex–mortality effect estimate and explained none of the between-study heterogeneity. While this analysis cannot exclude residual confounding by age within studies, it suggests that the observed age differences do not drive the variability in crude mortality estimates across studies.

This review has several strengths. We conducted a comprehensive search across multiple databases, followed established methodological standards, and included a large, diverse sample spanning different countries, age groups, and TBI severities. The large sample allowed us to investigate subgroup effects by age, severity, and TBI type, and to explore study-level age imbalance with meta-regression. We quantified heterogeneity using both *I*^2^ and prediction intervals, and assessed robustness through leave-one-out analyses, which together provide a transparent view of the uncertainty and variability in the underlying evidence.

Nonetheless, several limitations must be acknowledged. All included studies were observational in nature, making them susceptible to unmeasured factors. Factors such as socioeconomic status, access to care, pre-injury comorbidities, use of antithrombotic agents, and hormonal status (e.g., menstrual cycle phase, contraceptive use, or hormonal supplementation) were rarely reported and could not be accounted for. Mortality timing and TBI severity definitions varied across studies [e.g., in-hospital vs. longer-term mortality; Glasgow Coma Scale (GCS) vs. Abbreviated Injury Scale (AIS)-based severity]; however, sensitivity analyses suggested that excluding non-in-hospital mortality did not change the main conclusions. Residual heterogeneity remained substantial in most pooled sensitivity and subgroup analyses, indicating that additional unmeasured differences in patient populations, care pathways, or health systems likely influence outcomes. Finally, publication bias cannot be fully ruled out despite a nonsignificant Egger’s test.

## Conclusions

There appears to be no singular overall relationship between sex and mortality following TBI. Rather than a uniform female disadvantage or advantage, the association appears context-dependent, varying by study setting, patient selection, injury characteristics, and analytic choices, with signals observed in certain subgroups (e.g., as high-quality studies, severe TBI, or studies conducted before the 2000s) being sensitive to the influence of individual studies. Future research should move beyond whether sex, in general, influences outcomes, but instead focus on when, for whom, and under what conditions sex influences outcome, with the aim of informing individualized risk stratification and, where appropriate, sex-tailored TBI management.

## Supplementary Information

Below is the link to the electronic supplementary material.

Supplementary file1 (DOCX 2865 KB)

## Data Availability

All data analyzed in this systematic review and meta-analysis, including extracted study-level data on patient characteristics, mortality rates, and subgroup variables, are available from the corresponding author upon reasonable request. This includes the data extraction sheets, coding for subgroup analyses, and any statistical code used for the meta-analyses. Access may be provided for academic and research purposes, and requests will be considered on a case-by-case basis to ensure appropriate use.

## Artificial Intelligence

Generative artificial intelligence (AI) (ChatGPT, OpenAI) was used during manuscript preparation to assist with readability, grammar, and concise rephrasing. It was not used to generate or interpret data, nor to produce figures or statistical results. The authors reviewed and validated all AI-assisted content and take full responsibility for the final manuscript.
