# Supplementary material for: Association Between Sex and Mortality After Traumatic Brain Injury: A Systematic Review and Meta-Analysis
Source: Neurocrit Care. 2026 Apr 24;45(1):395–415. doi: 10.1007/s12028-026-02521-3 (PMC13369724; doi:10.1007/s12028-026-02521-3)
Supplement: Supplementary file 1 — Supplementary file1 (DOCX 2865 KB) [file 12028_2026_2521_MOESM1_ESM.docx]

**Supplemental Material: Figures and Tables**

**Supplemental Figure 1. Funnel plot.**

Funnel plot of included studies.


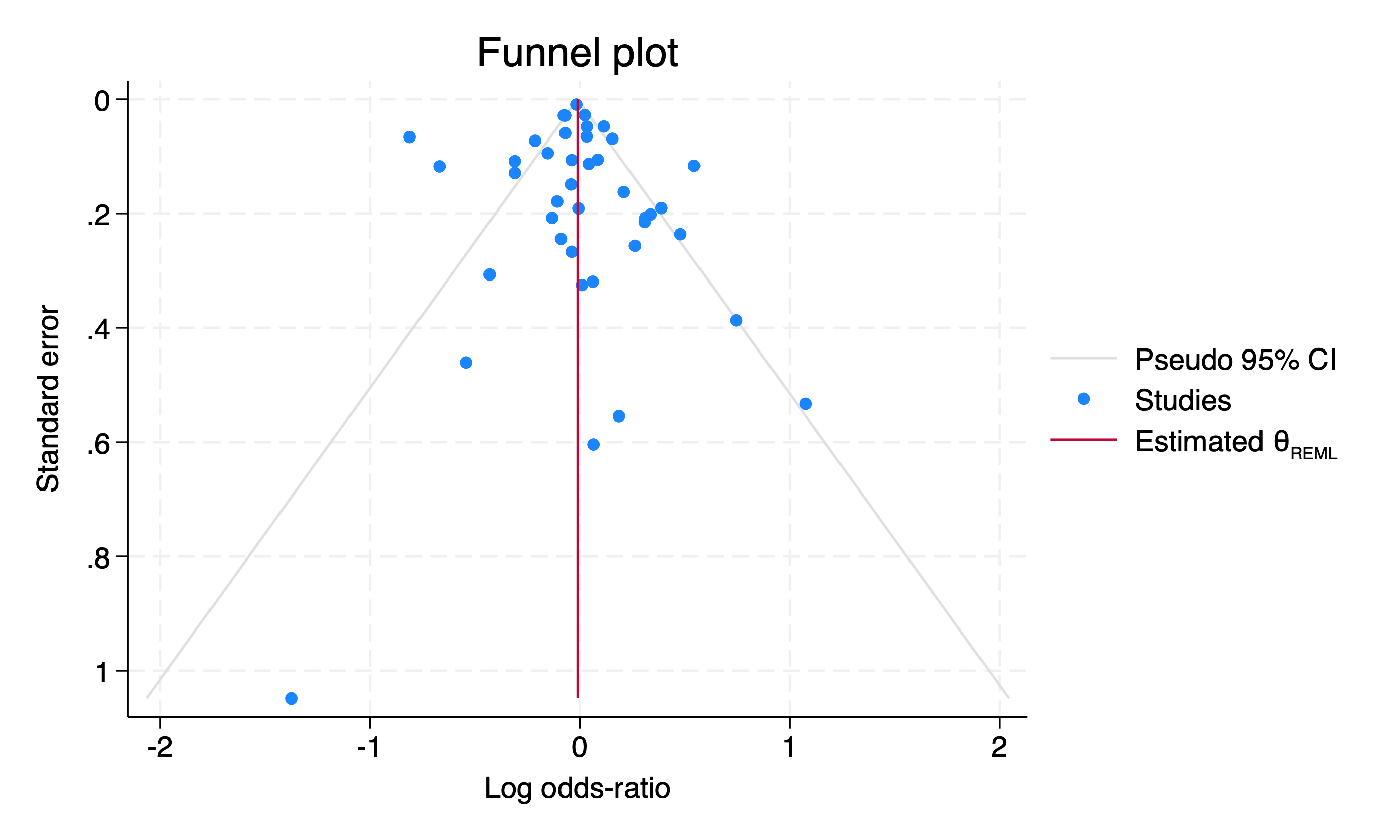
**Supplemental Figure 2. Forest plot, stratified on study quality.**

Forest plot summarizing the individual studies and pooled results of the meta-analysis, stratified for study quality.


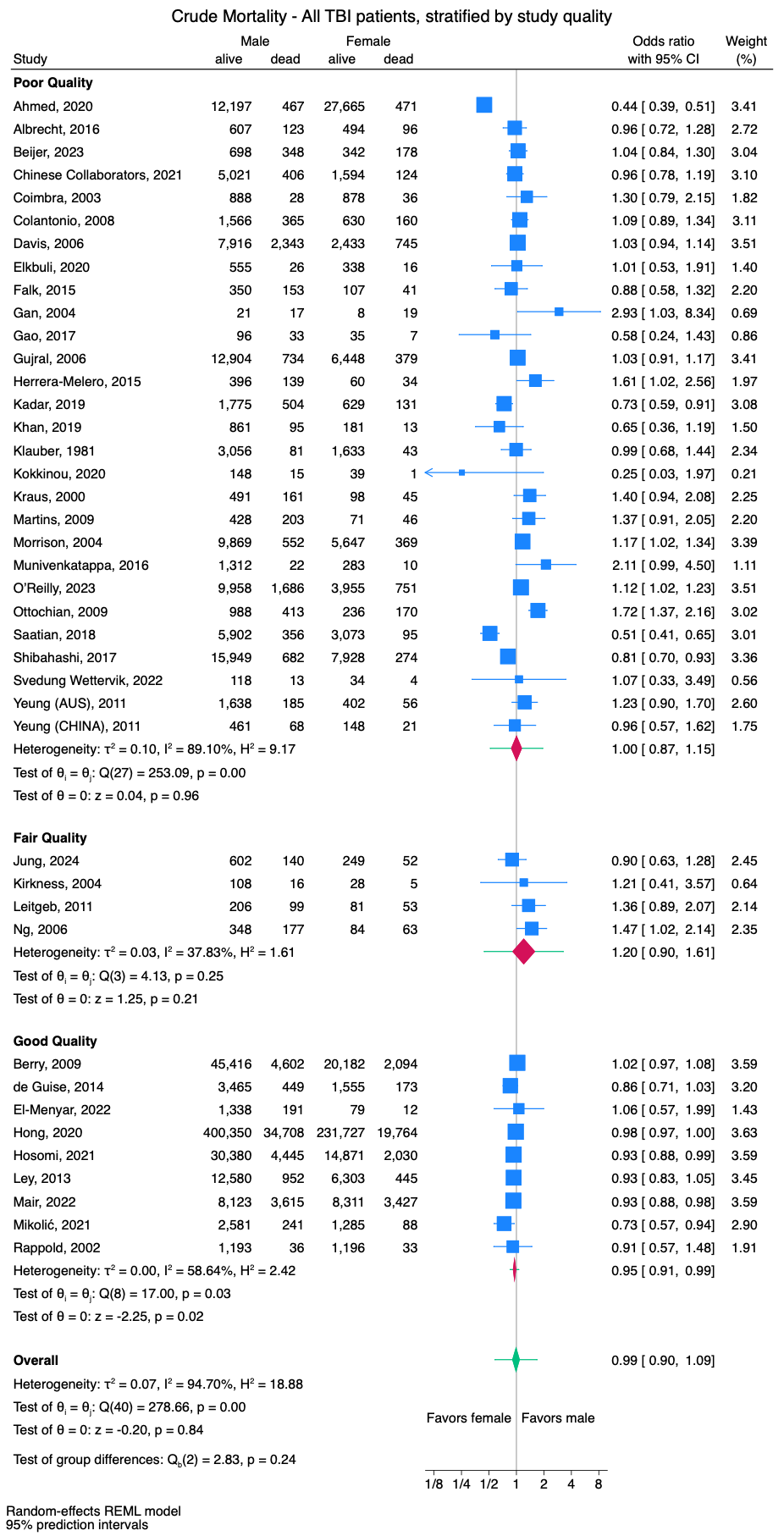


**Supplemental Figure 3. Forest plot, stratified on in-hospital mortality.**

Forest plot summarizing the individual studies and pooled results of the meta-analysis, stratified for in-hospital mortality.


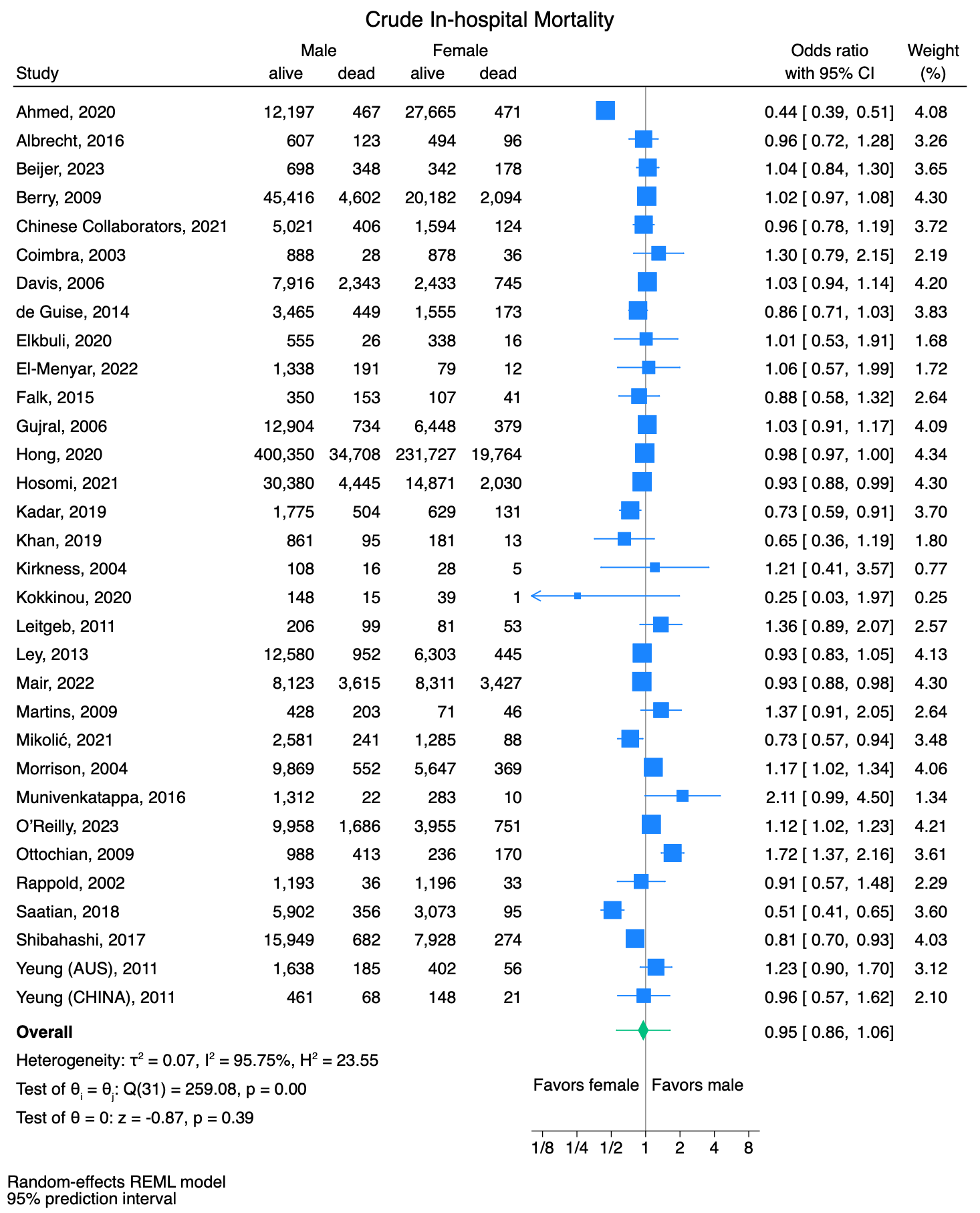


**Supplemental Figure 4. Forest plot, stratified on isolated TBI.**

Forest plot summarizing the individual studies and pooled results of the meta-analysis, stratified for isolated TBI.


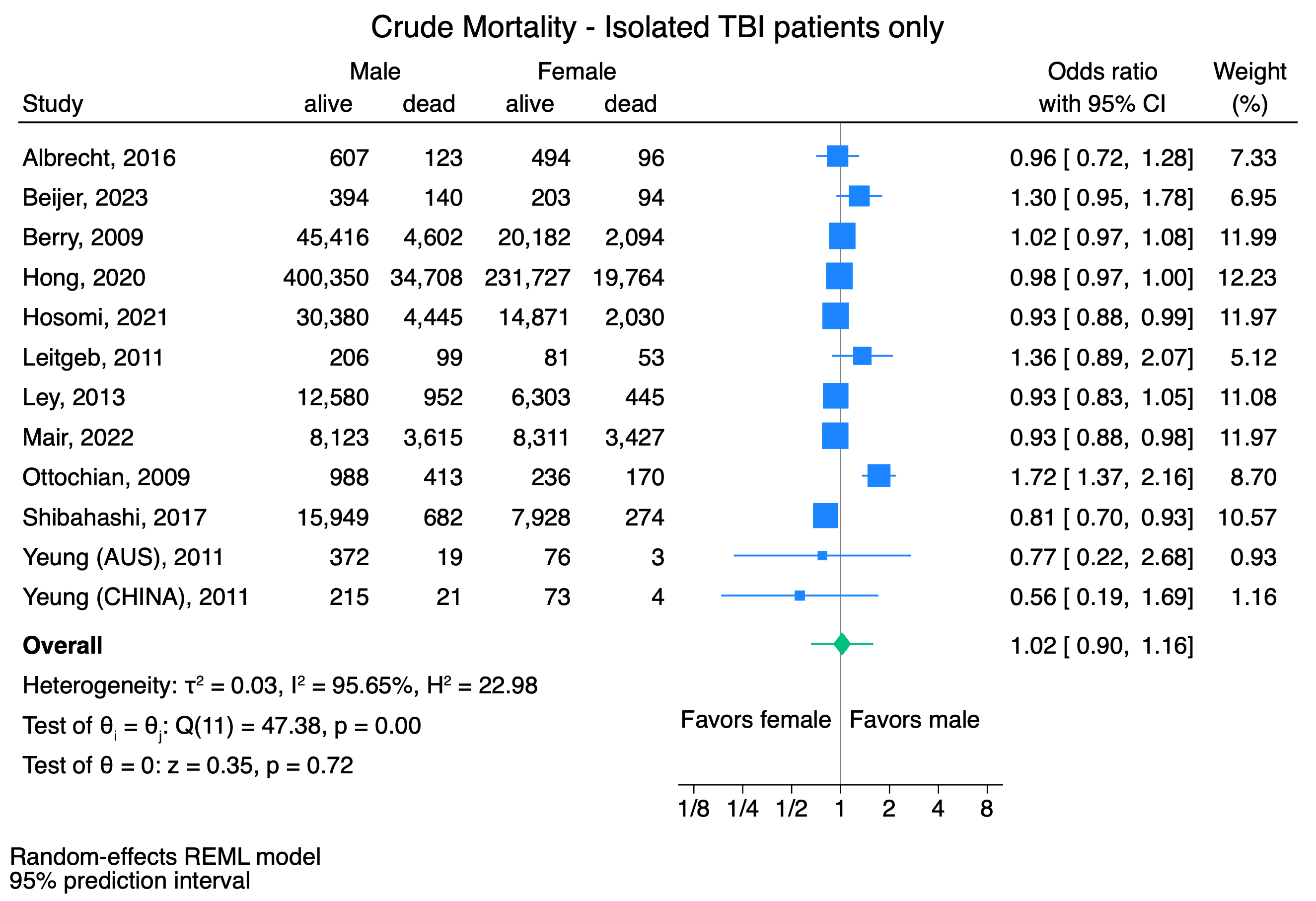


**Supplemental Figure 5. Forest plot, stratified on mixed TBI.**

Forest plot summarizing the individual studies and pooled results of the meta-analysis, stratified for mixed TBI.


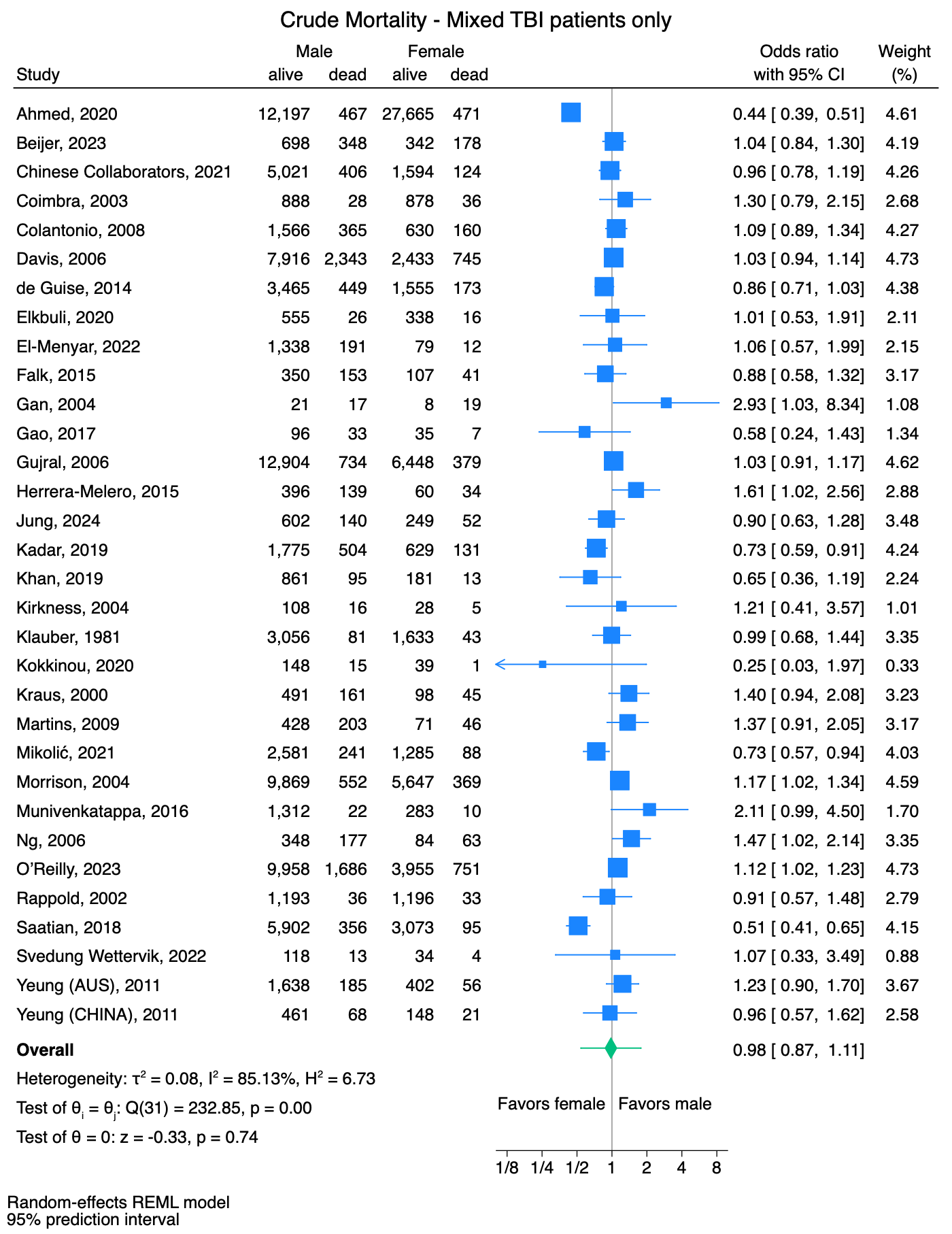


**Supplemental Figure 6. Forest plot, stratified on moderate-severe TBI.**

Forest plot summarizing the individual studies and pooled results of the meta-analysis, stratified for moderate-severe TBI.


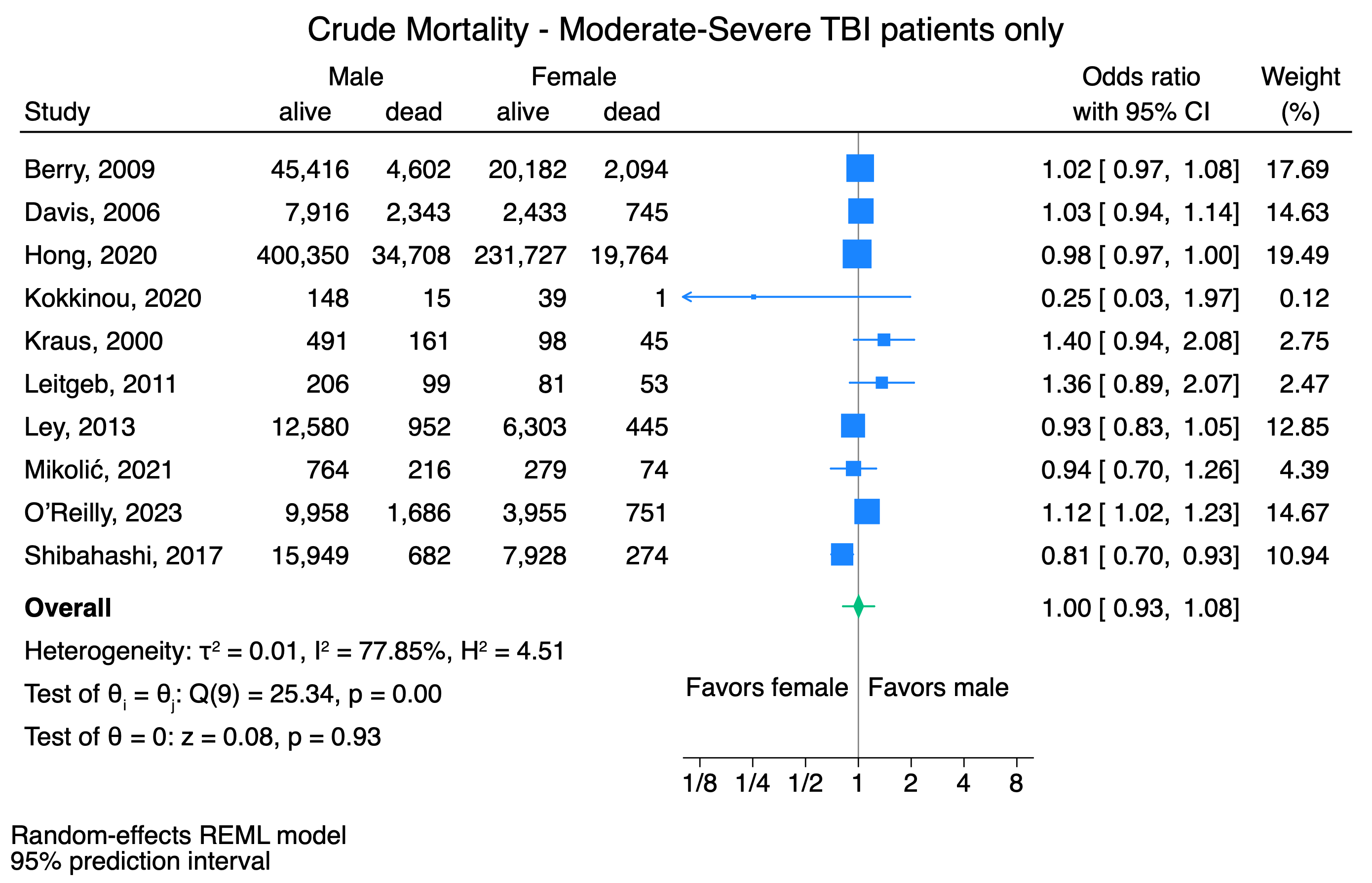


**Supplemental Figure 7. Forest plot, stratified on severe TBI.**

Forest plot summarizing the individual studies and pooled results of the meta-analysis, stratified for severe TBI.


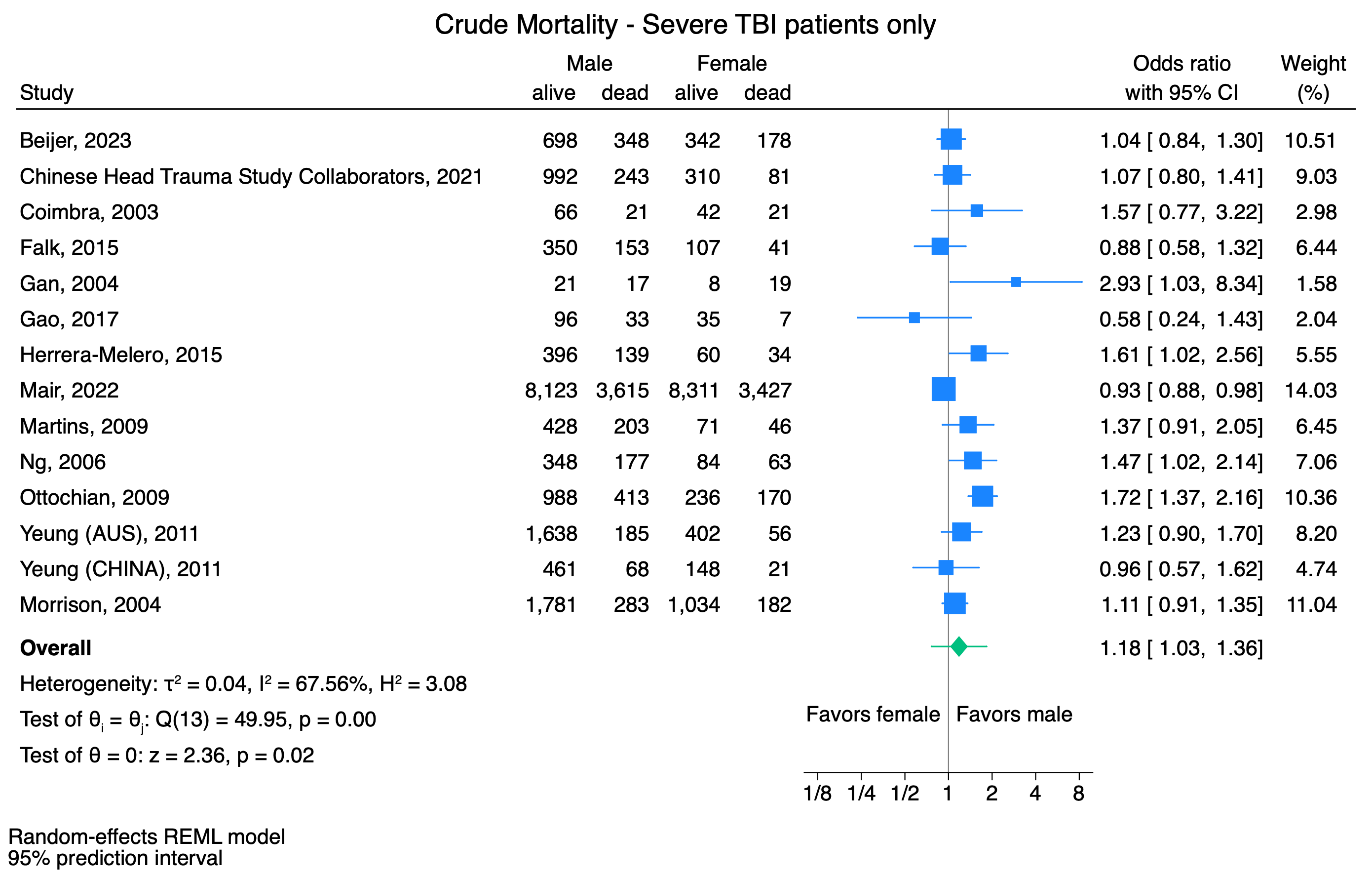


**Supplemental Figure 8. Forest plot, stratified on age.**

Forest plot summarizing the individual studies and pooled results of the meta-analysis, stratified for age.


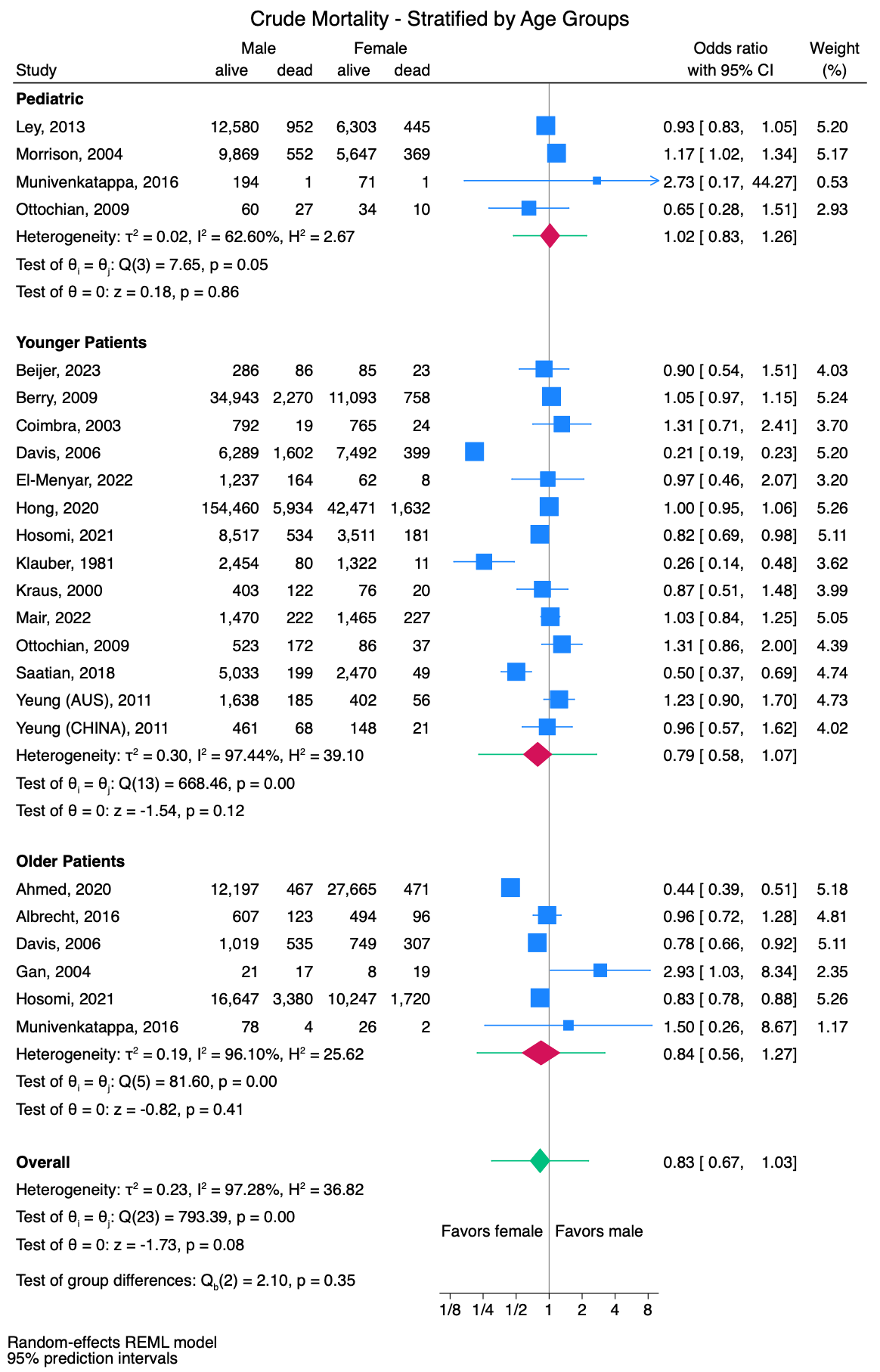


**Supplemental Figure 9. Forest plot, stratified on study period.**

Forest plot summarizing the individual studies and pooled results of the meta-analysis, stratified for study period.


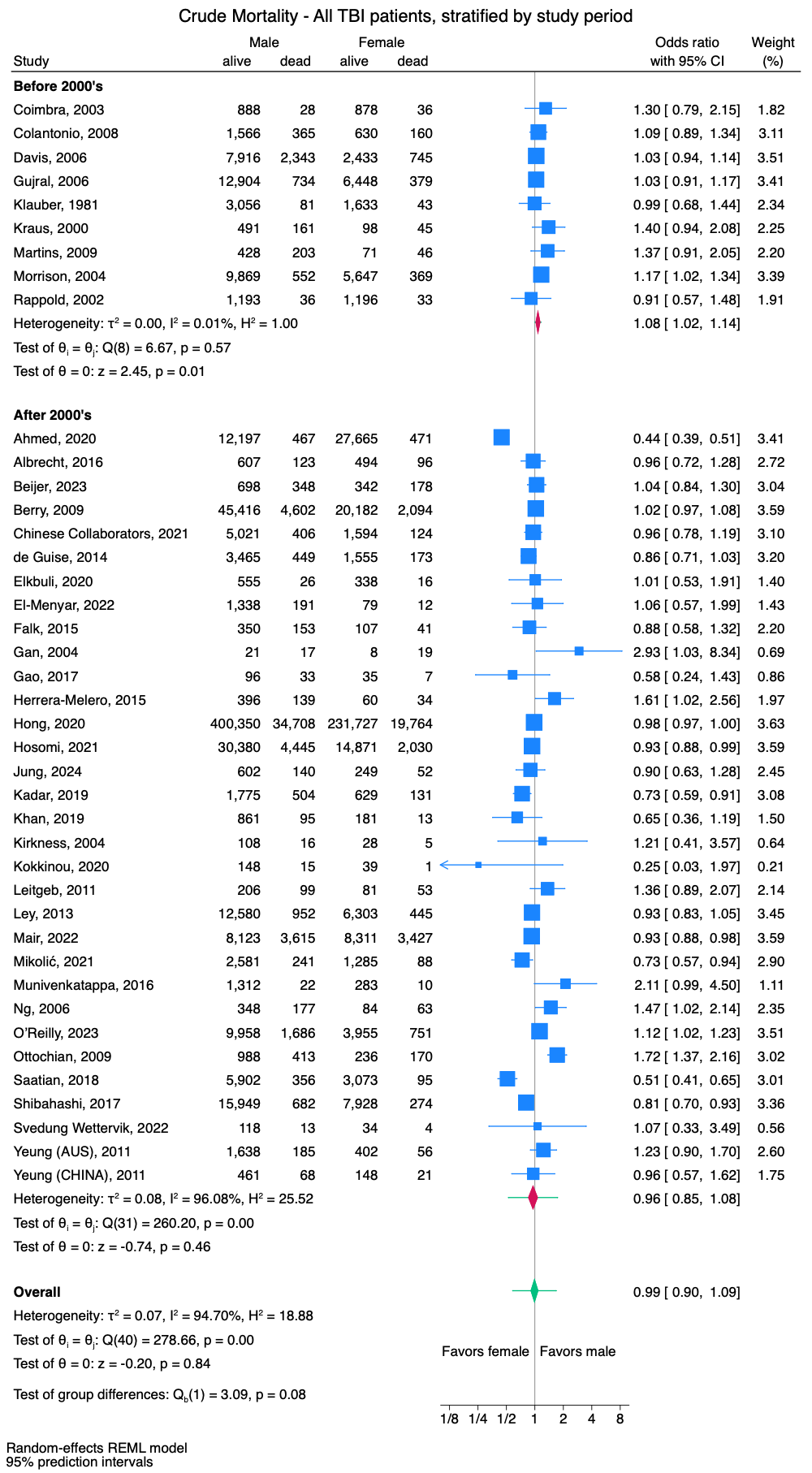


**Supplemental Table 1. General study characteristics**

| First Author | Year of publication | Country | Study design | Study in-/exclusion criteria | Study population | N female/male |
| --- | --- | --- | --- | --- | --- | --- |
| Ahmed | 2020 | USA | Retrospective study (NTDB) | 1Jan2012-31Dec2014  Geriatric (≥65 years) who experienced an unintentional ground level fall at home and were brought to the hospital, who suffered traumatic injury, and whose initial evaluation at the scene showed a normal SBP [Reference Range: 90-160mmHg), HR [Reference Range: 60-100 beats/min], and a GCS=15  Exclusion: patient records with missing or incomplete information for selected variables | TBI (mixed) | 28,136/12,664 |
| Albrecht | 2016 | USA | Retrospective cohort study | 1996-2012  Geriatric (≥65 years) patients with TBI (Head AIS>0) and with isolated TBI (defined as Head AIS>0 and Other Body Regions AIS=0) | TBI (mixed & isolated) | 590/730 (isolated) |
| Beijer | 2023 | Netherlands | Retrospective study (3 Level 1 Trauma Centers) | 1Jan2015-31Dec2018  Adult (>16years) patients with severe TBI (Head AIS>4)  Exclusion: patients with drowning, asphyxia, or burns | TBI (mixed & isolated) | 520/1,046 (overall)  108/372 (16-44y)  412/674 (>45y)  297/534 (isolated TBI)  40/172 (isolated TBI 16-44y)  257/362 (isolated TBI >45y) |
| Berry | 2009 | USA | Retrospective review (NTDB) | 2000-2005  Adult (>14years) patients with isolated moderate to severe TBI, defined as Head AIS>3 with AIS<3 for all other body regions (chest, abdomen, and extremity)  Exclusion: dead on arrival, dead within 24h, transferred out to another facility, admitted with a burn diagnosis, Head AIS>5, and any missing data including age, gender, and Head AIS | TBI (isolated) | 22,276/50,018 (overall)  11,851/37,213 (14-44y)  10,425/12,805 (>55y) |
| Chinese Head Trauma Study Collaborators | 2021 | China | Retrospective study (Databank, 47 centers) | 1Dec2008-20Aug2009  All patients with acute TBI (GCS3-15 assessed within 48h postinjury) who were admitted to the hospital | TBI (mixed) | 1,718/5,427 (overall)  391/1,235 (severe TBI) |
| Coimbra | 2003 | USA | Retrospective case-controlled study (Single Level 1 Trauma Center) | Jul1992-Dec1999  Adult (>18-64years) trauma patients sustaining TBI who were hospitalized for more than 24h, who were admitted to the intermediate care unit or to the surgical ICU, who died, or who were interfacility transfers to the UCSD Level I trauma center were entered into the trauma registry and considered for this analysis  Exclusion: those not sustaining TBI | TBI (mixed) | 914/916 (all GCS)  789/811 (all GCS <50y)  788/769 (GCS13-15)  683/680 (GCS13-15 <50y)  40/42 (GCS9-12)  36/36 (GCS9-12 <50y)  63/87 (GCS<9)  52/80 (GCS<9 <50y) |
| Colantonio | 2008 | Canada | Retrospective cohort study (OTR, 12 Specialized Lead Trauma Centers) | 1Apr1993-31Mar1995 (follow-up until 31Dec2002)  Adult (>15years) patients who sustained TBI (Head/Neck AIS<3=mild, 3=moderate, >3 severe) and had ISS>12 with ICD-9 codes for head injury (800, 801, 803-804, and 850-854) | TBI (mixed & isolated) | 790/1,931 (TBI) |
| Davis | 2006 | USA | Retrospective, registry-based analysis (5 Trauma Centers) | 1987-2003  Adult (>15years) patients with moderate-to-severe TBI (Head/Neck AIS>3), admitted for at least 24h  Exclusion: patients whose Head/Neck AIS was defined by a neck injury | TBI (mixed) | 3,178/10,259 (overall)  1,881/7,891 (15-49y)  1,297/2,368 (>50y)  411/1,411 (15-19y)  638/3,277 (20-29y)  463/1,933 (30-39y)  369/1,270 (40-49y)  241/814 (50-59y)  252/556 (60-69y)  804/998 (>70y) |
| de Guise | 2014 | Canada | Retrospective study | 2000-2011  All TBI patients | TBI (mixed) | 1,728/3,914 |
| Elkbuli | 2020 | USA | Retrospective review (Single Level 1 Trauma Center) | 2014-2017  All patients who experienced TBI | TBI (mixed) | 354/581 |
| El-Menyar | 2022 | Qatar | Retrospective analysis (Single Level 1 Trauma Center) | Jan2014-Feb2019  Adult (>14years) patients admitted due to TBI  Exclusion: dead on arrival, and dead within 24h or transferred from or to other facility | TBI (mixed) | 91/1,529 (overall)  70/1,401 (14-54y)  20/128 (>55y) |
| Falk | 2015 | Sweden | Retrospective study (Database) | 2000-2010  Adult (>15 years) patients with severe TBI with GCS<8 measured on admission to hospital and requiring neuro-intensive care | TBI (mixed) | 148/503 |
| Gan | 2004 | Singapore | Retrospective review of prospectively collected data (Single Center) | Aug1999-Jul2001  All patients with severe TBI | TBI (mixed) | 27/38 (>64y)  19/129 (20-40y) |
| Gao | 2017 | UK | Retrospective study | 2002-2012  All patients admitted to the Neurosciences and Trauma Critical Care Unit after severe head injury (GCS<8), with at least 72h recording of ICP, MAP and HR  Exclusion: those with more than 2h gaps and over 24h from ictus at the time of admission | TBI (mixed) | 42/129 |
| Gujral | 2006 | USA | Retrospective study | 1994-1998  All Colorado resident patients who were hospitalized or died from TBI (ICD-9: 800–801.9, 803–804.9 or 850–854.1, 959.01) | TBI (mixed) | 6,827/13,638 (overall)  5,973/11,114 (blunt)  854/2,524 (penetrating) |
| Herrera-Melero | 2015 | Spain | Retrospective cohort study (Single Center) | 1Jan2005-31Dec2012  Adult (>18years) patients with severe TBI (energy force transferred to the head; GCS score <8 after initial reanimation (indicating severe injury); and discarding being under the effects of drugs or alcohol  Exclusion: GCS =3 after initial reanimation and without the effects of sedative agents; cardiopulmonary resuscitation after the accident; unequivocal signs of brain death at admission; females undergoing cross-gender hormonal treatment; and pregnancy | TBI (mixed) | 94/535 |
| Hong | 2020 | USA | Retrospective analysis (NTDB) | Jan2007-Dec2016  Adult (>16years) blunt injury patients with isolated moderate to severe TBI (Head AIS>2=moderate to severe)  Exclusion: patients with mild TBI (Head AIS<2) and polytrauma patients with AIS>2 in other body regions | TBI (isolated, blunt) | 251,491/435,058 (overall)  44,103/160,394 (<45y)  23,655/65,442 (45-55y)  183,733/209,222 (>55y) |
| Hosomi | 2021 | Japan | Retrospective cohort study (JTDB, 272 Level 1 Trauma Centers) | Jan2004-Dec2018  All patients transported to the hospital for treatment for TBI (injury to the brain due to an external force)  Exclusion: Polytrauma defined as AIS>3 for all other body regions (chest, abdomen, and extremity), Head AIS=6 (lethal injury) or 9 (unspecified injury), cardiopulmonary arrest on hospital arrival (SBP 0mmHg and/or heart rate of 0bpm), or missing data for variables required for logistic regression analysis | TBI (isolated) | 16,901/34,825 (overall)  3,692/9,051 (<50y)  13,209/23,874 (>50y)  11,967/20,027 (>60y)  672/1,403 (0-9y)  850/2,273 (10-19y)  694/287 (20-29y)  626/2,162 (30-39y)  850/2,926 (40-49y)  1,242/3,847 (50-59y)  2,609/6,282 (60-69y)  4,047/7,259 (70-79y)  4,189/5,596 (80-89y)  1,089/873 (90-99y)  33/17 (>100y) |
| Jung | 2024 | Korea | Multicenter prospective cohort study (5 Academic Centers) | Dec2018-Mar2023  Adult (>18years) patients with TBI (diagnosis of intracranial injury confirmed by CT or MRI scans conducted in the ED, including those with diffuse axial injuries and intracranial hemorrhages) who were admitted to the ED of collaborating hospitals via EMS within 72h of injury  Exclusion: patients with unknown information on long-term functional outcomes or obesity status | TBI (mixed) | 301/742 |
| Kadar | 2019 | USA | Retrospective study (Trauma Registry) | 2004-2012  All patients treated for TBI (GCS 13-15=mild, 9-12=moderate, 3-8=severe) and admitted to the trauma service (patients with head injury with persistent unconsciousness or focal signs such as seizures; posturing or the inability to respond to simple commands; transmediastinal gunshot wounds; spinal cord injury with paralysis; maternal trauma with significant mechanism and/or obvious trauma at 20-32 weeks gestation; pediatric trauma including blunt or penetrating head, chest, or abdominal trauma; for blunt or penetrating trauma, patients with unstable vital signs (SBP<90mmHg, RR<10/>29breath/min, head injury with GCS <10) (including patients that were dead on arrival or died in the ED)  Exclusion: patients that died on the scene and were not transported to a trauma center, and patients who were treated in the ED and discharged in less than 12h | TBI (mixed) | 760/2,279 |
| Khan | 2019 | India | Prospective observational study (Single Level 1 Trauma Center) | Jul2016-Dec2017  All patients admitted with TBI  Exclusion: preexisting severe neurologic disorders | TBI (mixed) | 194/956 (overall)  145/794 (adult)  49/162 (pediatric)  140/593 (mild TBI)  29/176 (moderate TBI)  25/187 (severe TBI) |
| Kirkness | 2004 | USA | Prospective study (Single Level 1 Trauma Center) | 23months study period  Adult (>16years) patients with TBI or trauma-related subarachnoid hemorrhage who were admitted to the ICU, with intracranial pressure and cerebral perfusion pressure monitoring  Exclusion: bilateral fixed pupils and impending death at the time individuals were being screened for potential study enrollment | TBI (mixed) | 33/124 |
| Klauber | 1981 | USA | Prospective study (10 Centers selected from 30 Centers) | 1978  All head-injured patients admitted to the hospital (ICD-8: codes 800, 801, 805, 806, 850-854)  Exclusion: neck injury in the absence of spinal cord injury | TBI (mixed) | 1,676/3,137 (all injuries)  430/877 (selected hospitals)  1,333/2,534 (all injuries <40y)  343/601 (all injuries >40y)  139/217 (all injuries 0-9y)  100/188 (selected hospitals 0-9y)  525/1,049 (all injuries 10-19y)  87/255 (selected hospitals 10-19y)  669/1,268 (all injuries 20-39y)  121/305 (selected hospitals 20-39y)  196/307 (all injuries 40-59y)  51/71 (selected hospitals 40-59y)  147/294 (all injuries >60y)  71/58 (selected hospitals >60y) |
| Kokkinou | 2020 | Cyprus | Observational national cohort study (Single Level 1 Trauma Center) | Jan2013-Dec2016  Adult patients with moderate-to-severe TBI (GCS 9-12=moderate, GCS3-8=severe) admitted in the ICU with at least 6-months follow-up  Exclusion: mild TBI, hospital admission over 24h after injury | TBI (mixed) | 40/163 |
| Kraus | 2000 | USA | Prospective cohort study (2 Centers) | Dec1992-Jul1996  Adult (>16 years) TBI patients (traumatic insult to the brain including diffuse axonal injury, contusion, laceration, intracranial hemorrhage, and acute intracranial hematoma (epidural/subdural) with GCS 3-12 in the ED after stabilization (generally from 4-6h post injury)  Exclusion: injury limited to only olfactory, optic, or facial nerves, or to the spinal cord at/below C2-3, severe preexisting comorbidity: terminal illness at time of trauma, history of chronic severe neurological disturbance or diminished mental capacity, severe brain hypoxia | TBI (mixed) | 143/652 (overall)  49/285 (16-29y)  47/240 (30-49y)  47/127 (>50y) |
| Leitgeb | 2011 | Austria | Prospective study (17 Centers) | Mar2002-Jun2005 & Mar2009-Apr2010  All Austrian patients with isolated (no additional injury with AIS>2) moderate and severe TBI  Exclusion: death before admission to the ICU | TBI (isolated) | 134/305 |
| Ley | 2013 | USA | Retrospective review (NTDB) | 2007-2008  Pediatric (<18years) patients who required admission for blunt trauma, with an Head AIS>3 (moderate-to-severe TBI)  Exclusion: all patients with AIS>3 for any other body region, dead on arrival, with AIS=6 for any region, or missing data | TBI (isolated, blunt) | 6,748/13,532 (overall)  3,865/6,270 (0-12y)  2,883/7,262 (13-18y) |
| Mair | 2022 | Germany | Multicenter prospective database (TraumaRegister DGU, >700 Centers) | 2009-2020  Adult (>18years) patients with severe TBI (Head AIS>3) and concomitant injuries with a severity of AIS<3 in German speaking countries  Exclusion: pregnant females, patients with missing essential information (sex, GCS, blood pressure at scene). All patients who were transferred to the reporting hospital or who were transferred out after initial treatment | TBI (isolated) | 11,738/11,738 (matched)  1,692/1,692 (<45y matched)  10,046/10,046 (>45y matched) |
| Martins | 2009 | Brazil | Prospective study (Single Center) | 1Jan1994-31Dec2003  All patients with severe TBI (GCS <8 after acute neurosurgical resuscitation, or deterioration to that level within 48h of impact) admitted to the ICU  Exclusion: victims of gunshot injury and patients who evolved to brain death before 24h of admission | TBI (mixed) | 117/631 |
| Mikolić | 2021 | Europe | Prospective multi-center longitudinal observational study (CENTER-TBI, 63 European Centers) | Dec2014-Dec2017  All patients with clinical diagnosis of TBI (GCS13-15 mild, GCS3-12 moderate-severe) and were presented to a study center <24h of injury either to the ED, admission ward or ICU, had an indication for CT scanning, and provided informed consent  Exclusion: any severe pre-existing neurological disorder that could confound outcome assessments | TBI (mixed) | 1,373/2,822 (overall)  1,020/1,842 (mild TBI)  353/980 (moderate-severe TBI) |
| Morrison | 2004 | USA | Retrospective study (NPTR) | Apr1994-Sep2000  Pediatric patients with nonpenetrating TBI (ICD-9 codes 800.xx–801.xx, 803.xx, and 850.xx–854.xx, which include the diagnoses skull fracture, concussion, intracranial hemorrhage (subarachnoid, subdural, extradural, and other), and unspecified intracranial injury) and multiple trauma including TBI  Exclusion: penetrating TBI | TBI (mixed, blunt) | 6,016/10,421 (overall)  1,314/2,114 (0-7y mild)  1,288/1,853 (0-7y moderate)  660/920 (0-7y severe)  87/141 (0-7y life-threatening)  524/1,095 (8-12y mild)  444/905 (8-12y moderate)  300/555 (8-12y severe)  56/104 (8-12y life-threatening)  478/925 (13-19y mild)  403/860 (13-19y moderate)  256/589 (13-19y severe)  77/130 (13-19y life-threatening) |
| Munivenkatappa | 2016 | India | Retrospective study (Single Tertiary Center) | 1Jan2010-15Mar2010  All patients who presented with TBI | TBI (mixed) | 293/1,334 (overall)  72/195 (<18y)  193/1,057 (19-60y)  28/82 (>61y) |
| Ng | 2006 | Singapore | Prospective study | Apr1999-Dec2004  All patients admitted with severe TBI (post resuscitation GCS<8) to a tertiary care neurocritical unit | TBI (mixed) | 147/525 |
| O’Reilly | 2023 | Australia & New Zealand | Retrospective study (Trauma Registry, 23 Centers) | 1Jul2015-30Jun2020  All patients with moderate to severe TBI (defined by Head AIS>2), with ISS>12 or who died in hospital  Exclusion: cases for which key variable data (age, sex, death in hospital) were incomplete | TBI (mixed) | 4,706/11,644 |
| Ottochian | 2009 | USA | Retrospective review (Single Center) | 1Jan1998-31Dec2005  All blunt trauma patients with isolated severe TBI (Head AIS>3, with an AIS<3 for all other body regions (chest, abdomen, extremity)) admitted to the hospital  Exclusion: all patients who were transferred to another acute care facility before 1 week, Head AIS=6 | TBI (isolated, blunt) | 406/1,401 (overall)  44/87 (<14y)  123/695 (14-44y)  55/235 (45-54y)  184/384 (>55y) |
| Rappold | 2002 | USA | Retrospective case-controlled study | Jul1992-Dec1999  Adult (18-64 years) trauma patients with blunt trauma, and with/without closed head injury and/or with/without shock (SBP<90/>90), hospitalized for more than 3 days, or admitted to the ICU, or died, or were interfacility transfers | TBI (mixed, blunt) | 1,229/1,229 (overall, matched)  867/867 (TBI, SBP>90, matched)  49/49 (TBI, SBP<90, matched) |
| Saatian | 2018 | Iran | Retrospective registry based study (Single Center) | Mar2013-Dec2016  All patients with TBI (defined as ICD-10 codes for head and neck injuries)  Exclusion: non-traumatic head and neck codes. Incomplete data | TBI (mixed) | 3,168/6,258 (overall)  2,519/5,232 (<50y)  649/1,026 (>50y)  877/1,309 (0-10y)  309/896 (11-20y)  575/1,493 (21-30y)  451/961 (31-40y)  307/573 (41-50y)  268/447 (51-60y)  176/263 (61-70y)  135/199 (71-80y)  62/95 (81-90y)  8/21 (91-100y)  0/1 (101-100y) |
| Shibahashi | 2017 | Japan | Retrospective study (NJTDB, 260 Centers) | 2004-2015  Adult (>16years) patients who talked (defined as GCS verbal score >3 on admission) after TBI (defined as Head AIS3-5)  Exclusion: SBP<40mmHg, or presence of severe injury (AIS>3) on other body regions | TBI (isolated) | 8,202/16,631 |
| Svedung Wettervik | 2022 | Sweden | Retrospective study (Single Center) | 2008-2020  Adult (>16years) patients with TBI treated at the neurointensive care unit monitored with both ICDP and cerebral microdialysis with standardized treatment protocol (Treatment goals were ICP <20mmHg, CPP>60mmHg, systolic blood pressure >100 mmHg, pO2 >12 kPa, blood glucose 5–10 mmol/L, electrolytes within normal ranges, normovolemia, and body temperature <38)  Exclusion: early brain death | TBI (mixed) | 38/131 |
| Yeung | 2011 | Australia & China | Retrospective study (Trauma Registry, Australia: 139 Centers, China: 2 Centers) | 1Jan2001-31Dec2007  **Australia**: Adult (12-45years, goals: including only premenopausal females) trauma patients with an Head AIS>3 irrespective of other major injuries (from the trauma registry included patients with any of the following: death due to injury, an ISS>15, ICU LOS >24h requiring MV, or urgent surgery)  Exclusion: patients with minor head injury and those with a loss of consciousness without brain hemorrhage or undisplaced closed skull fracture  **China**: Adult (12-45years, goals: including only premenopausal females) trauma patients with an Head AIS>3 irrespective of other major injuries (from the trauma registry included patients with any of the following: death due to injury, patients triaged as “critical” or “emergency” in the ED (triage categories 1 and 2), all ICU admissions, or major trauma patients transferred from another acute hospital)  Exclusion: patients with minor head injury and those with a loss of consciousness without brain hemorrhage or undisplaced closed skull fracture | TBI (mixed & isolated) | 458/1,823 (Australia)  79/391 (Australia isolated TBI)  169/529 (China)  77/236 (China isolated TBI) |

AIS: Abbreviated Injury Scale, CT: Computed Tomography, DGU: Deutsche Gesellschaft fur Unfallchirurgie, ED: Emergency Department, GCS: Glasgow Coma Scale, h: hours, HR: Heartrate, ICD: International Classification of Diseases, ICP: Intracranial Pressure, ICU: Intensive Care Unit, ISS: Injury Severity Score, kph: kilometer per hour, LOS: Length Of Stay, m: meter, MAP: Mean Arterial Pressure, min: minutes, MV: Mechanical Ventilation, NJTDB: National Japan Trauma Data Bank, NPTR: National Pediatric Trauma Registry, NTDB: National Trauma Data Bank, OTR: Ontario Trauma Registry, RR: Respiratory Rate, RTS: Revised Trauma Score, SBP: Systolic Blood Pressure, SCISR: Spinal Cord Injury Surveillance Registry, SI: Shock Index, TBI: Traumatic Brain Injury, y: years of age

**Supplemental Table 2. Age, ISS and mechanism (organized as female/male)**

| *First Author* | *Year of publication* | *Age in years (mean±SD or median(IQR))* | *P-value* | *ISS (mean±SD or median(IQR)) and/or other scores for injury severity* | *P-value* | *Mechanism (blunt or penetrating)* | *P-value* | *Conclusion (X=no differences)* |
| --- | --- | --- | --- | --- | --- | --- | --- | --- |
| Ahmed | 2020 | NR | P=NR | NR | P=NR | NR | P=NR | NR |
| Albrecht | 2016 | 78.9±7.7/76.8±7.5 | P<0.001 | **AIS Head (groups 1, 2, 3, 4, >5)** 20%/20%, 4%/2%, 13%/10%, 38%/42%, 25%/27% | P=0.12 | Blunt 587/709 (99%/97%)  Penetrating 1/13 (<1%/2%) | P=0.005 | Females ↑age ↑blunt |
| Beijer | 2023 | *Overall:* 65(16-98)/56(16-95)  *16-44y:* 25(16-44)/28(16-44)  *>45y:* 70(45-98)/68(45-95)  *Isolated TBI overall*: 68(16-98)/58(16-95)  *Isolated TBI 16-44y*: 25(16-44)/30(16-44)  *Isolated TBI >45y*: 71(45-98)/68(45-95) | P<0.001  P=0.001  P=0.008  P<0.001  P=0.014  P=0.024 | *Overall:* 25(16-75)/26(16-75)  *Overall*:  **AIS Neck>3** 0.6%/1.3%  **AIS Spine>3** 4.0%/6.6%  **AIS Thorax>3** 17.3%/26.5%  **AIS Abdomen>3** 2.5%/2.8%  **AIS Lower extremities>3** 6.7%/7.2%  **RTS** 5.97(0-7.84)/5.97(0-7.84)  *16-44y:* 26(16-75)/29(16-75)  *16-44y*:  **AIS Neck>3** 0.9%/2.4%  **AIS Spine>3** 10.2%/7.5%  **AIS Thorax>3** 28.7%/31.2%  **AIS Abdomen>3** 10.2%/6.2%  **AIS Lower extremities>3** 15.7%/10.8%  **RTS** 4.09(0-7.84)/5.03(0-7.84)  *>45y:* 25(16-59)/25(16-75)  *>45y*:  **AIS Neck>3** 0.5%/0.7%  **AIS Spine>3** 2.4%/6.1%  **AIS Thorax>3** 14.3%/23.9%  **AIS Abdomen>3** 0.5%/0.9%  **AIS Lower extremities>3** 4.4%/5.2%  **RTS** 6.76(0-7.84)/5.97(1.76-7.84)  *Isolated TBI overall*: 25(16-75)/25(16-75)  **RTS** 6.07(0-7.84)/6.90(1.76-7.84)  *Isolated TBI 16-44y*: 23(16-75)/24(16-75)  **RTS** 5.97(4.09-7.84)/6.90(2.63-7.84)  *Isolated TBI >45y*: 25(16-33)/25(16-75)  **RTS** 6.75(0-7.84)/6.90(1.76-7.84) | P<0.001  P=0.171  P=0.041  P<0.001  P=0.753  P=0.749  P=0.005  P=0.395  P=0.339  P=0.373  P=0.623  P=0.153  P=0.158  P=0.650  P=0.006  P=0.716  P=0.006  P<0.001  P=0.717  P=0.541  P=0.007  P=0.807  P=0.986  P=0.202  P=0.612  P=0.826  P=0.765 | *Overall*: blunt 98.0%/97.6%  *16-44y*: blunt 98.0%/97.6%  *>45y:* blunt 99.8%/97.8%  *Isolated TBI overall*: blunt 99.7%/97.2%  *Isolated TBI 16-44y*: blunt 97.5%/98.3%  *Isolated TBI >45y*: blunt 99.2%/96.6% | P=0.098  P=0.345  P=0.008  P=0.013  P=0.570  P=0.003 | *Overall:* Females ↑age ↓ISS ↓AIS Spine>3 ↓AIS Thorax>3 ↑RTS  *16-44y:* Females ↓age  *>45y:* Females ↑age ↓ISS ↓AIS Spine>3 ↓AIS Thorax>3 ↑blunt ↑RTS  *Isolated TBI overall*: Females ↑age ↑blunt  *Isolated TBI 16-44y*: Females ↓age  *Isolated TBI >45y*: Females ↑age ↑blunt |
| Berry | 2009 | 51.0±20.1/41.9±20.1 | P<0.0001 | 21.3±9.7/21.8±9.7 | P<0.0001 | Blunt 98.4%/96.2% | P<0.0001 | Females ↑age ↓ISS ↑blunt |
| Chinese Head Trauma Study Collaborators | 2021 | 41.1±2.0/40.4±2.3 | P>0.05 | NR | P=NR | NR | P=NR | X |
| Coimbra | 2003 | *All GCS:* 35.6/34.1  *All GCS <50y:* NR  *GCS13-15:* NR  *GCS13-15 <50y:* NR  *GCS9-12:* NR  *GCS9-12 <50y:* NR  *GCS<9:* NR  *GCS<9 <50y:* NR | P=ns  P=ns  P=NR  P=NR  P=NR  P=NR  P=NR  P=NR | *All GCS:* 11.8/11.8  **RTS** 7.50/7.42  *All GCS <50y:* 11.3/11.8  **RTS** 7.52/7.41  *GCS13-15:* 9.7/9.8  **RTS** 7.8/7.8  *GCS13-15 <50y:* 9.4/9.7  **RTS** 7.8/7.8  *GCS9-12:* 17.6/11.6  **RTS** 6.73/6.8  *GCS9-12 <50y:* 16.8/11.4  **RTS** 6.7/6.8  *GCS<9:* 29.4/26.0  **RTS** 4.1/4.3  *GCS<9 <50y:* 28.8/26.0  **RTS** 4.2/4.3 | P=ns  P=ns  P=ns  P=ns  P=ns  P=ns  P=ns  P=ns  P=ns  P=ns  P=ns  P=ns  P=ns  P=ns  P=ns  P=ns | *All GCS:* NR  *All GCS <50y:* NR  *GCS13-15:* NR  *GCS13-15 <50y:* NR  *GCS9-12:* NR  *GCS9-12 <50y:* NR  *GCS<9:* NR  *GCS<9 <50y:* NR | P=ns  P=ns  P= NR  P= NR  P= NR  P= NR  P= NR  P= NR | *All GCS:* X  *All GCS <50y:* X  *GCS13-15:* X  *GCS13-15 <50y:* X  *GCS9-12:* X  *GCS9-12 <50y:* X  *GCS<9:* X  *GCS<9 <50y:* X |
| Colantonio | 2008 | NR | P=NR | NR | P=NR | NR | P=NR | NR |
| Davis | 2006 | NR | P=NR | NR | P=NR | NR | P=NR | NR |
| de Guise | 2014 | 59.5(35.3-79.0)/47.9(30.0-66.6) | P<0.001 | 24(16-27)/25(17-29) | P=0.001 | NR | P=NR | Females ↑age ↓ISS |
| Elkbuli | 2020 | 76(49.25-84)/53(30-77) | P=0.002 | 16(10-22)/17(10-25)  **Head AIS** 3(3-4)/4(3-4) | P>0.05  P>0.05 | 98.3%/95.2% | P>0.05 | Females ↑age |
| El-Menyar | 2022 | *Overall:* 39.9±18.5/34.1±13.5  *14-54y*: NR  *>55y*: NR | P=0.004  P=NR  P=NR | *Overall:* 19(13-27)/22(14-29)  *Overall:* **AIS Head** 3(3-4)/3(3-5)  *14-54y*: 22(14-29)/22(14-29)  *14-54y*: **AIS Head** 3(3-5)/3(3-5)  *>55y*: 17(10-20)/19(12-29)  *>55y*: **AIS Head** 3(3-4)/4(3-5) | P=0.55  P=0.44  P=ns  P=ns  P=ns  P=ns | *Overall:* NR  *14-54y*: NR  *>55y*: NR | P=NR  P=NR  P=NR | *Overall:* Females ↑age  *14-54y*: X  *>55y*: X |
| Falk | 2015 | 47.8/47.8 | P=0.53 | NR | P=NR | NR | P=NR | X |
| Gan | 2004 | NR | P=NR | NR | P=NR | NR | P=NR | NR |
| Gao | 2017 | NR (overall cohort: 38.1±15) | P=NR | NR | P=NR | NR | P=NR | NR |
| Gujral | 2006 | NR | P=NR | NR | P=NR | Blunt 87.5%/81.5% | P<0.05 | Females ↑blunt |
| Herrera-Melero | 2015 | 36(23-59.2)/31(23-47) | P=0.051 | 26(25-34)/26(25-34)  **RTS** 9(8-10)/10(8-10) | P=0.495  P=0.062 | NR | P=NR | X |
| Hong | 2020 | *<45y:* 28.6±8.72/28.6±8.27  *45-55y:* 50.44±3.13/50.22±3.13  *>55y:* 77.91±9.95/73.83±10.6 | P=0.020  P<0.001  P<0.001 | *<45y:* 15.54±6.67/15.94±6.56  *45-55y:* 15.69±6.35/16.33±6.48  *>55y:* 15.91±6.04/16.49±6.36 | P<0.001  P<0.001  P<0.001 | N/A (all blunt) | N/A (all blunt) | *<45y:* Females ↑age ↓ISS  *45-55y:* Females ↑age ↓ISS  *>55y:* Females ↑age ↓ISS |
| Hosomi | 2021 | 72(54-82)/64(42-77) | P<0.001 | **Max Head AIS** 4(3-4)/4(3-4) | P<0.001 | Penetrating 0.1%/0.1% | P=NR | Females ↑age ↓Max Head AIS |
| Jung | 2024 | 18-49y 9%/22.4%  50-59y 11.3%/18.3%  60-69y 25.2%/23.3%  70-79y 30.2%/23.7%  >80y 24.3%/12.3% | P<0.001 | **NISS**  1-8 13%/14.3%  9-15 34.9%/31.8%  16-24 27.6%/26%  25-75 24.6%/27.9% | P=0.283 | NR | P=NR | Females ↑age |
| Kadar | 2019 | NR (overall cohort: 43±24) | P=NR | NR | P=NR | NR | P=NR | NR |
| Khan | 2019 | NR (adult: 36.37±15.80)  NR (pediatric: 6.65±3.84) | P=NR  P=NR | NR | P=NR | NR | P=NR | NR |
| Kirkness | 2004 | 39.9±20.7/36.3±17.4 | P=0.374 | 30.1±9.1/28.6±9.8  **Head AIS=5** 52%/31% | P=0.230  P=0.039 | NR | P=NR | Females ↑Head AIS=5 |
| Klauber | 1981 | NR | P=ns | NR | P=0.011 | NR | P=NR | Females ↑ISS |
| Kokkinou | 2020 | 53.18±22.44/43.90±21.11 | P<0.05 | NR | P>0.05 | NR | P=NR | Females ↑age |
| Kraus | 2000 | NR (32.9% women >50years, 19.5% men >50years) | P<0.001 | NR | P=NR | Blunt 90.2%/85.3%  Penetrating 9.8%/14.7% | P=NR | Females ↑age |
| Leitgeb | 2011 | 61.4±22.6/50.4±21.5 | P<0.001 | 23.2±17.9/21.5±13.7 | P=0.36 | Blunt 94.8%/94.1%  Penetrating 5.2%/5.9% | P=NR | Females ↑age |
| Ley | 2013 | *0-12y:* 4.9±3.9/5.5±4.1  *13-18y:* 16±1.6/16.1±1.6 | P<0.0001  P=0.098 | *0-12y:* 16.6±7.1/16.4±7.3  **Head AIS** 4(3-4)/4(304)  *13-18y:* 20.3±9.2/19.5±8.7  **Head AIS** 4(3-4)/4(3-4) | P=0.37  P=0.80  P=0.0003  P=0.0003 | N/A (all blunt) | N/A (all blunt) | *0-12y:* Females ↓age  *13-18y:* Females ↑ISS |
| Mair | 2022 | 67.5±19.6/66.7±19.1 | P=NR | 21.3±8.1/21.6±8.2 | P=NR | Penetrating 1.1%/2.3% | P<0.001 | Females ↑blunt |
| Martins | 2009 | NR (overall cohort: 34.8±16.3) | P=NR | NR | P=NR | NR | P=NR | NR |
| Mikolić | 2021 | *Mild TBI:* 58(37-73)/50(32-65)  *Moderate TBI:* 53(30-69)/47(29-64) | P<0.001  P=0.02 | *Mild TBI:* 9(4-16)/13(8-19)  *Moderate TBI:* 33(25-43)/34(25-48) | <0.00  P=0.28 | NR | P=NR | *Mild TBI:* Females ↑age ↓ISS  *Moderate-severe TBI:* Females ↑age |
| Morrison | 2004 | NR | P=sig | NR | P=ns | N/A (all blunt) | N/A (all blunt) | Females ↓age |
| Munivenkatappa | 2016 | *Overall:* 35.06/33.67 | P=0.212 | NR | P=NR | NR | P=NR | X |
| Ng | 2006 | 53.7±22.8/43.0±18.6 | P<0.001 | **Multiple injuries** 25.2%/25.1% | P=0.995 | NR | P=NR | Females ↑age |
| O’Reilly | 2023 | NR (overall cohort: 50.5±26.1) | P=NR | NR | P=NR | NR (overall cohort: blunt 98.1%) | P=NR | NR |
| Ottochian | 2009 | 50.8±26.6/43.0±20.7 | P<0.00001 | 24.6±6.9/23.2±6.9 | P<0.0001 | N/A (all blunt) | N/A (all blunt) | Females ↑age ↑ISS |
| Rappold | 2002 | *Overall*: 36.6/34.9 (mean)  *TBI&SBP>90:* 35.4/34.1 (mean)  *TBI&*SBP<90: 38.6/34.0 (mean) | P=ns  P=ns | *Overall*: 15.6/16.3 (mean)  *TBI&*SBP>90: 10.9/10.8  *TBI&*SBP>90: **AIS head** 2.2/2.3 (mean)  *TBI&*SBP>90: **AIS chest** 2.6/2.6 (mean)  *TBI&*SBP>90: **AIS abdominal** 2.5/2.5 (mean)  *TBI&*SBP>90*:* **Admission RTS** 7.6/7.54 (mean)  *TBI&*SBP<90: 27.7/28.4 (mean)  *TBI&*SBP<90: **AIS head** 3.3/3.4 (mean)  *TBI&*SBP<90: **AIS chest** 3.0/3.4  *TBI&*SBP<90: **AIS abdominal** 3.2/2.9  *TBI&*SBP<90*:* **Admission RTS** 5.2/5.2 (mean) | P=ns  P=ns  P=ns  P=ns  P=ns  P=ns  P=ns  P=ns  P=ns  P=ns  P=ns | N/A (all blunt) | N/A (all blunt) | *Overall:* X  *TBI&SBP>90:* X  *TBI&*SBP<90: X |
| Saatian | 2018 | NR (overall cohort: 29.70±21.46) | P=NR | NR | P=NR | NR | P=NR | NR |
| Shibahashi | 2017 | NR | P=NR | NR | P=NR | NR | P=NR | NR |
| Svedung Wettervik | 2022 | 41(20-59)/49(32-59) | P=0.13 | NR | P=NR | NR | P=NR | X |
| Yeung | 2011 | *Australia*: 26.3±9.3/27.2±8.7  *China*: 27.9±9.7/29.6±9.6 | P=0.04  P=0.049 | *Australia*: **ISS groups (<16, 16-25, 26-40, >40)** 5.0%/4.3%, 41.0%/45.3%, 34.3%/33.7%, 19.7%/16.7%  *Australia*: **Isolated TBI vs multiple trauma** 17.2%/21.4%, 82.8%/78.6%  *Australia*: **Multiple-system injury** 82.8%/78.6%  *Australia*: **Severe abdominal injuries** NR  *Australia*: **AIS Head** NR  *Australia*: **AIS Neck** NR  *Australia*: **AIS Face** NR  *Australia*: **AIS Thorax** NR  *Australia*: **AIS Extremities** NR  *Australia*: **AIS External regions** NR  *China*: **ISS groups (<16, 16-25, 26-40, >40)** 20.1%/20.6%, 36.7%/40.1%, 28.4%/24.8%, 14.8%/14.6%  *China*: **Isolated TBI vs multiple trauma** 45.6%/44.6%, 54.4%/55.4%  *China*: **Multiple-system injury** 54.4%/55.2%  *China*: **Severe abdominal injuries** NR  *China*: **AIS Head** NR  *China*: **AIS Neck** NR  *China*: **AIS Face** NR  *China*: **AIS Thorax** NR  *China*: **AIS Extremities** NR  *China*: **AIS External regions** NR | P=0.30  P=0.047  P=0.047  P<0.001  P=ns  P=ns  P=ns  P=ns  P=ns  P=ns  P=0.79  P=0.83  P=0.86  P=0.046  P=ns  P=ns  P=ns  P=ns  P=ns  P=ns | *Australia*: NR  *China*: NR | P=NR  P=NR | *Australia*: Females ↓age ↑multiple trauma ↑multiple-system injury ↓severe abdominal injuries  *China*: Females ↓age ↓severe abdominal injuries |

AIS: Abbreviated Injury Scale, ICISS: International Classification of Disease based Injury Severity Score, ISS: Injury Severity Score, NISS: New Injury Severity Score, NR: Not Reported, RTS: Revised Trauma Score, y: years of age

**Appendix A**

| **Search** | **PubMed Query – June 4, 2025** | **Results** |
| --- | --- | --- |
| #1 | "Sex Factors"[Mesh] OR "Sex Characteristics"[Mesh] OR "Sex Distribution"[Mesh] OR "sex inequalit*"[tiab] OR "sex bias"[tiab] OR "sex diff*"[tiab] OR "sex dimorph*"[tiab] OR "sex-specific*"[tiab] OR "sex based"[tiab] OR "sex disparit*"[tiab] OR "sex factor*"[tiab] OR "sex characteristic*"[tiab] OR "sex dichotom*"[tiab] OR "sex related"[tiab] OR "gender inequalit*"[tiab] OR "gender bias"[tiab] OR "gender diff*"[tiab] OR "gender dimorph*"[tiab] OR "gender specific*"[tiab] OR "gender based"[tiab] OR "gender disparit*"[tiab] OR "gender factor*"[tiab] OR "gender characteristic*"[tiab] OR "gender dichotom*"[tiab] OR "gender related"[tiab] OR "male versus female"[tiab] OR "men versus women"[tiab] OR "men and women"[tiab] OR "women and men"[tiab] OR "male and female"[tiab] OR "female and male"[tiab] | 636,311 |
| #2 | "Shock, Hemorrhagic"[Mesh] OR "Hypovolemia"[Mesh] OR "Trauma Centers"[Mesh] OR "trauma center*"[tiab] OR "trauma centre*"[tiab] OR "trauma regist*"[tiab] OR "hypovolem*"[tiab] OR "severe trauma*"[tiab] OR "severely injured"[tiab] OR "massive transfusion"[tiab] OR (("hemorrhag*"[tiab] OR "haemorrhag*"[tiab] OR "bleeding*"[tiab] OR "blood loss"[tiab]) AND ("shock"[tiab] OR "trauma"[tiab] OR traumatic*[tiab] OR "incident"[tiab] OR "injur*"[tiab] OR "accident"[tiab])) | 154,225 |
| #3 | "Mortality"[Mesh] OR "Hemorrhage/mortality"[Mesh] OR "Wounds and Injuries/mortality"[Mesh] OR "mortalit*"[tiab] OR "survival outcome"[tiab] OR "survival rate*"[tiab] OR "death rate*"[tiab] OR "case fatality rate*"[tiab] | 1,574,364 |
| #4 | #1 AND #2 AND #3 | 1,060 |
| #5 | #4 NOT ("Animals"[Mesh] NOT "Humans"[Mesh]) | 1,027 |

| **Search** | **Embase.com Query – June 4, 2025** | **Results** |
| --- | --- | --- |
| #1 | 'sex ratio'/exp OR 'sex difference'/exp OR ((('sex' OR 'gender') NEAR/3 ('inequalit*' OR 'bias' OR 'diff*' OR 'dimorph*' OR 'specific*' OR 'based' OR 'disparit*' OR 'factor*' OR 'characteristic*' OR 'dichotom*' OR 'related')):ti,ab,kw) OR ((('male' OR 'males' OR 'men' OR 'man') NEAR/3 ('female' OR 'females' OR 'woman' OR 'women')):ti,ab,kw) | 1,609,749 |
| #2 | 'hemorrhagic shock'/exp OR 'hypovolemia'/exp OR 'hospital emergency service'/exp OR 'hypovolem*':ti,ab,kw OR 'trauma center*':ti,ab,kw OR 'trauma centre*' OR 'trauma regist*' OR 'severe trauma*':ti,ab,kw OR 'severely injured':ti,ab,kw OR 'massive transfusion':ti,ab,kw OR ((('hemorrhag*' OR 'haemorrhag*' OR 'bleeding' OR 'blood') NEAR/3 ('shock' OR 'trauma' OR 'incident' OR 'injur*' OR 'accident')):ti,ab,kw) | 151,217 |
| #3 | 'mortality'/exp OR 'hospital mortality'/exp OR 'mortality rate'/exp OR 'mortalit*':ti,ab,kw OR 'survival outcome':ti,ab,kw OR 'survival rate*':ti,ab,kw OR 'death rate*':ti,ab,kw OR 'case fatality rate*':ti,ab,kw | 2,476,478 |
| #4 | #1 AND #2 AND #3 | 2,299 |
| #5 | #4 NOT ([animals]/lim NOT [humans]/lim) | 2,261 |
| #6 | #5 NOT ('clinical trial'/it OR 'conference abstract'/it OR 'conference review'/it) | 1,704 |

| **Search** | **Clarivate Analytics/Web of Science Core Collection Query – June 4, 2025  *Indexes=SCI-EXPANDED, SSCI, A&HCI, ESCI Timespan=All years*** | **Results** |
| --- | --- | --- |
| #1 | TS=((("sex" OR "gender") NEAR/3 ("inequalit*" OR "bias" OR "diff*" OR "dimorph*" OR "specific*" OR "based" OR "disparit*" OR "factor*" OR "characteristic*" OR "dichotom*" OR "related") ) OR (("male" OR "males" OR "men" OR "man") NEAR/3 ("female" OR "females" OR "woman" OR "women") )) | 1,194,844 |
| #2 | TS=("hypovolem*" OR "trauma center*" OR "trauma centre*" OR "trauma regist*" OR "severe trauma*" OR "severely injured" OR "massive transfusion" OR (("hemorrhag*" OR "haemorrhag*" OR "bleeding" OR "blood") NEAR/3 ("shock" OR "trauma" OR "incident" OR "injur*" OR "accident"))) | 95,917 |
| #3 | TS= ("mortalit*" OR "survival outcome" OR "survival rate*" OR "death rate*" OR "case fatality rate*") | 1,749,777 |
| #4 | #1 AND #2 AND #3 | 1,197 |

| **Search** | **Wiley/Cochrane Library Query – June 4, 2025** | **Results** |
| --- | --- | --- |
| #1 | ((sex NEXT inequalit*) OR "sex bias" OR (sex NEXT diff*) OR (sex NEXT dimorph*) OR (sex NEXT specific*) OR "sex based" OR (sex NEXT disparit*) OR (sex NEXT factor*) OR (sex NEXT characteristic*) OR (sex NEXT dichotom*) OR "sex related" OR (gender NEXT inequalit*) OR "gender bias" OR (gender NEXT diff*) OR (gender NEXT dimorph*) OR (gender NEXT specific*) OR "gender based" OR (gender NEXT disparit*) OR (gender NEXT factor*) OR (gender NEXT characteristic*) OR (gender NEXT dichotom*) OR "gender related" OR "male versus female" OR "men versus women" OR "men and women" OR "women and men" OR "male and female" OR "female and male") :ti,ab,kw (word variations have been searched) | 51975 |
| #2 | (hypovolem* OR (trauma NEXT center*) OR (trauma NEXT centre*) OR (trauma NEXT regist*) OR (severe NEXT trauma*) OR "severely injured" OR "massive transfusion" OR (("hemorrhag*" OR "haemorrhag*" OR "bleeding" OR "blood loss") AND ("shock" OR "trauma" OR "incident" OR injur* OR "accident"))) :ti,ab,kw (word variations have been searched) | 32450 |
| #3 | (mortalit* OR ("survival" NEXT (outcome OR rate*)) OR (death NEXT rate*) OR (case NEXT fatality NEXT rate*)) :ti,ab,kw (word variations have been searched) | 145796 |
| #4 | #1 AND #2 AND #3 | 161 |

| **Search** | **Google Scholar – Sep 6, 2025** | **Results** |
| --- | --- | --- |
|  | Severe trauma\|severe injury\|hemorrhagic shock+ ”sex\|gender+differences\|dimorphism\|dichotomy\|specific\|characteristics\|factor\|bias\|inequalities\|disparities”+mortality\|survival rate\|death rate | In accordance with the protocol, the first 200 hits were analyzed |

**Appendix B**

| **Assessment of quality of a cohort study – Newcastle Ottawa Scale (NOS)** |
| --- |
| **Selection maximum 4 stars** |
| 1. Representativeness of the female cohort (with respect to the male cohort)  a) truly representative = ☆  b) somewhat representative = ☆  c) selected group = 0  d) no description of the derivation of the cohort = 0 |
| 2. Selection of the female cohort  a) drawn from the same community as the male cohort = ☆  b) drawn from a different source = 0  c) no description of the derivation of the non intervention cohort = 0 |
| 3. Ascertainment of sex (female or male)  a) secure record (eg surgical record) = ☆  b) structured interview = ☆  c) written self report = 0  d) other / no description = 0 |
| 4. Demonstration that mortality was not present at start of study  a) yes = ☆  b) no = 0 |
| **Comparability maximum 2 stars*** |
| 1. Comparability of cohorts on the basis of the design or analysis: studies reported baseline values of females and males regarding to age, injury severity score, mechanism of injury  a) 3 out of 3 = ☆☆  b) 2 out of 3 = ☆  c) 0-1 out of 3 = 0 |
| **Outcome maximum 3 stars** |
| 1. Assessment of mortality  a) independent blind assessment = ☆  b) record linkage = ☆  c) self report = 0  d) other / no description = 0 |
| 2. Was follow up long enough for mortality to occur  a) yes = ☆ (in-hospital mortality: minimal follow up until discharge, 30-day mortality: 30 days etc.)  b) no = 0 |
| 3. Adequacy of follow up of cohorts  a) complete follow up: all subjects accounted for = ☆  b) subjects lost to follow up unlikely to introduce bias: number lost <= 20%, = ☆  or description of those lost suggesting no different from those followed  c) follow up rate < 80% (select an adequate %) and no description of those lost = 0  d) no statement = 0 |
| **Size maximum 1 star*** |
| 1. Size of the cohort  a) >1.000 patients in each group (female and male) = ☆  b) <1.000 patients in each group (female and male) = 0 |
| **Cohort design maximum 1 star*** |
| 1. Design of the study  a) Prospective cohort = ☆  b) Retrospective cohort (trauma registry) = 0 |
| ** edited fields compared to standard NOS* |

| **Thresholds for converting the NOS to AHRQ standards [https://www.ncbi.nlm.nih.gov/books/NBK115843/bin/appe-fm3.pdf]** |
| --- |
| **Good quality** |
| - 3 or 4 stars in selection domain - 1 or 2 stars in comparability domain - 2 or 3 stars in outcome domain - 1 star in the size domain* - 0 or 1 star in the cohort design domain* |
| **Fair quality** |
| - 2 stars in selection domain - 1 star in comparability domain - 2 or 3 stars in outcome domain - 1 star in the size/cohort design domains combined* |
| **Poor quality** |
| - 0 or 1 star in selection domain - 0 stars in comparability domain - 0 or 1 stars in outcome domain - 0 stars in the size/cohort design domains combined |
| ** edited fields compared to standard NOS* |
